# An early cell shape transition drives evolutionary expansion of the human forebrain

**DOI:** 10.1016/j.cell.2021.02.050

**Published:** 2021-04-15

**Authors:** Silvia Benito-Kwiecinski, Stefano L. Giandomenico, Magdalena Sutcliffe, Erlend S. Riis, Paula Freire-Pritchett, Iva Kelava, Stephanie Wunderlich, Ulrich Martin, Gregory A. Wray, Kate McDole, Madeline A. Lancaster

**Affiliations:** 1 MRC Laboratory of Molecular Biology, Cambridge Biomedical Campus, Francis Crick Avenue, Cambridge CB2 0QH, UK; 2 Department of Applied Mathematics and Theoretical Physics, University of Cambridge, Wilberforce Road, Cambridge CB3 0WA, UK; 3 Leibniz Research Laboratories for Biotechnology and Artificial Organs (LEBAO), REBIRTH-Research Center for Translational and Regenerative Medicine, Hannover Medical School, 30625 Hannover, Germany; 4 Biomedical Research in Endstage and Obstructive Lung Disease (BREATH), Member of the German Center for Lung Research (DZL), Hannover Medical School, 30625 Hannover, Germany; 5 Department of Biology, Duke University, Biological Sciences Building, 124 Science Drive, Durham, NC 27708, USA

**Keywords:** brain, evolution, cell shape, organoids, brain expansion, neuroepithelium, neural stem cells, ZEB2, gorilla, chimpanzee

## Abstract

The human brain has undergone rapid expansion since humans diverged from other great apes, but the mechanism of this human-specific enlargement is still unknown. Here, we use cerebral organoids derived from human, gorilla, and chimpanzee cells to study developmental mechanisms driving evolutionary brain expansion. We find that neuroepithelial differentiation is a protracted process in apes, involving a previously unrecognized transition state characterized by a change in cell shape. Furthermore, we show that human organoids are larger due to a delay in this transition, associated with differences in interkinetic nuclear migration and cell cycle length. Comparative RNA sequencing (RNA-seq) reveals differences in expression dynamics of cell morphogenesis factors, including ZEB2, a known epithelial-mesenchymal transition regulator. We show that ZEB2 promotes neuroepithelial transition, and its manipulation and downstream signaling leads to acquisition of nonhuman ape architecture in the human context and vice versa, establishing an important role for neuroepithelial cell shape in human brain expansion.

## Highlights

• Human brain organoids are expanded relative to nonhuman apes prior to neurogenesis
• Ape neural progenitors go through a newly identified transition morphotype state
• Delayed morphological transition with shorter cell cycles underlie human expansion
• ZEB2 is as an evolutionary regulator of this transition

## Graphical abstract

**Figure:**
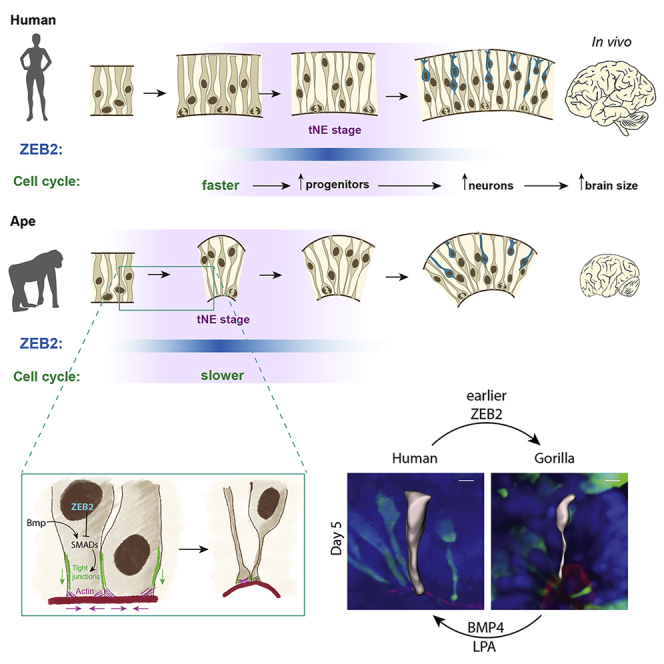

## Teaser

Cerebral organoid models reveal that differences in the duration of a developmental transitional state driven by the factor ZEB2 underlie the basis of brain expansion in humans in comparison to great apes.

## Introduction

One of the most distinctive features of humans as a species is an enlarged brain. The human brain exhibits a 1,000-fold increase in total neuron number compared with mice (Herculano-Houzel et al., 2006) and is the largest of all primates, roughly 3-fold larger than that of our closest living relatives, chimpanzees and gorillas (Herculano-Houzel, 2012). A number of studies have examined differences in brain development between human, mouse, and other distantly related model organisms (Bae et al., 2015; Lui et al., 2011; Sousa et al., 2017), highlighting divergence in neural progenitor behavior (Florio et al., 2015), neurogenesis (O’Neill et al., 2018), and cytoarchitecture (Dehay et al., 2015; Fietz et al., 2010; Hansen et al., 2010). Although much has been learned about the mechanisms governing evolutionary divergence of primates from rodents, human-specific changes compared to other apes are less well understood.

Comparative studies have led to a number of important insights. Early in development, before the onset of neurogenesis, the human forebrain is already larger than that of mouse and macaque, indicating differences in expansion of precursor cells called neuroepithelial (NE) cells (Rakic, 2007). Thus, it has long been hypothesized that changes in these cells could lead to an expansion of the neocortical primordium (Rakic, 1988, 1995). Comparison of adult brain morphology has also revealed non-uniform thickening of cortical layers in primates, especially apes (Charvet et al., 2017; DeFelipe et al., 2002; Hutsler et al., 2005; Nowakowski et al., 2016; Rockel et al., 1980; Sousa et al., 2017). Given that cortical layers are generated successively over time, this points to expansion events in humans and other apes after the onset of neurogenesis. However, comparisons of the human brain to that of other apes reveal a general increase in size without an expansion in particular layers of the cerebral cortex (Donahue et al., 2018, de Sousa et al., 2010; Hutsler et al., 2005), suggesting that while primate expansion may have been governed by both early and late changes, human-specific differences likely take place prior to the generation of cortical layer neurons. However, without being able to examine and manipulate early progenitor cell behavior in apes and humans, these observations have remained correlative.

Most of our understanding of the principles governing mammalian brain development comes from studying the mouse, where at ∼E10, cells rapidly undergo a switch from NE cells to radial glia (RG), triggering the onset of neurogenesis (Kriegstein and Alvarez-Buylla, 2009). Before this transition, NE cells exhibit a columnar morphology and divide in a symmetric proliferative manner. This proliferation results in an exponential expansion leading to a ballooning out of the neocortex and enlargement of the ventricles in humans (Bayer et al., 1993). NE cells are strongly epithelial in character and held together near their apical surface by cell-cell junctions, namely tight junction components (i.e., OCLN) and adherens junction components (i.e., CDH1) (Aaku-Saraste et al., 1996; Rogers et al., 2018). In rodents and other model vertebrates, it is well-documented that the transition to neurogenic RG cells involves a loss of some epithelial features, a thinning and elongation of the cell, the onset of expression of certain glial markers, and a switch to asymmetric cell division with one daughter remaining a RG, and the other becoming more differentiated and leaving the ventricular zone (Götz and Huttner, 2005). This switch in cell fate thus leads to a change in expansion from tangential to radial, with additional cells being added to the more basal layers, rather than along the apical surface of the ventricles. Although this NE-to-RG conversion has been studied in mice, where it occurs in a matter of hours, it remains entirely unknown how this process occurs in humans and other apes.

The ability to generate brain organoids from induced pluripotent stem cells (iPSCs) has enabled researchers to study a variety of neurodevelopmental processes that were previously inaccessible (Lancaster et al., 2013; Kadoshima et al., 2013; Paşca et al., 2015; Quadrato et al., 2017). Recently, a number of studies have started tackling evolutionary differences across primates, highlighting human-specific patterns of gene expression (Kanton et al., 2019; Mora-Bermúdez et al., 2016; Pollen et al., 2019). Human organoids appear to develop and mature at a slower pace, with neurogenesis commencing later (Otani et al., 2016), RG cells displaying a longer mitotic phase (Mora-Bermúdez et al., 2016), and neurons showing a decreased expression of genes related to neuronal maturation (Kanton et al., 2019). Similarly, genetic comparisons have identified a number of human specific loci capable of regulating RG expansion and neurogenesis (Fiddes et al., 2018; Suzuki et al., 2018; Boyd et al., 2015). Although these studies point to important differences in terms of RG behaviors, all these comparisons have been performed at stages where neurogenesis is already underway. Given the neuroanatomical comparisons that point to an early expansion and a universal increase in neurons, it would be highly informative to examine early human and ape progenitors at a stage preceding neurogenesis. Furthermore, extending comparative analysis to include other great apes beyond chimpanzee would help identify true human-specific cellular adaptations (rather than chimpanzee-specific differences) involved in brain size determination.

Here, we examined developing human, chimpanzee, and gorilla brain organoids. We found that during early NE expansion, the NE to RG transition in human and ape is a gradual process, taking place over the course of several days, unlike the rapid differentiation of NE to RG cells described in mouse, and it involves an intermediate cell morphology state we named transitioning NE (tNE) cells. During this transition, cell shape changes occur before the change in cell identity and the onset of neurogenesis. Furthermore, human NE cells are delayed relative to ape in making this transition and proliferate more. To identify the molecular mechanism underlying these differences, we performed time-resolved sequencing analysis and identified differential expression dynamics of the zinc-finger transcription factor *ZEB2*. Through *ZEB2* loss- and gain-of-function experiments, we show that this gene is a driver of the NE to RG cell transition. Finally, manipulation of *ZEB2* expression and its downstream signaling is able to force a human-like phenotype in the ape context and vice versa. Thus, our data suggest that in humans, a delayed onset of *ZEB2* expression extends the NE stage, contributing to human-specific neocortical expansion.

## Results

### Human telencephalic organoids show differences in tissue architecture prior to neurogenesis

**Figure S1:**
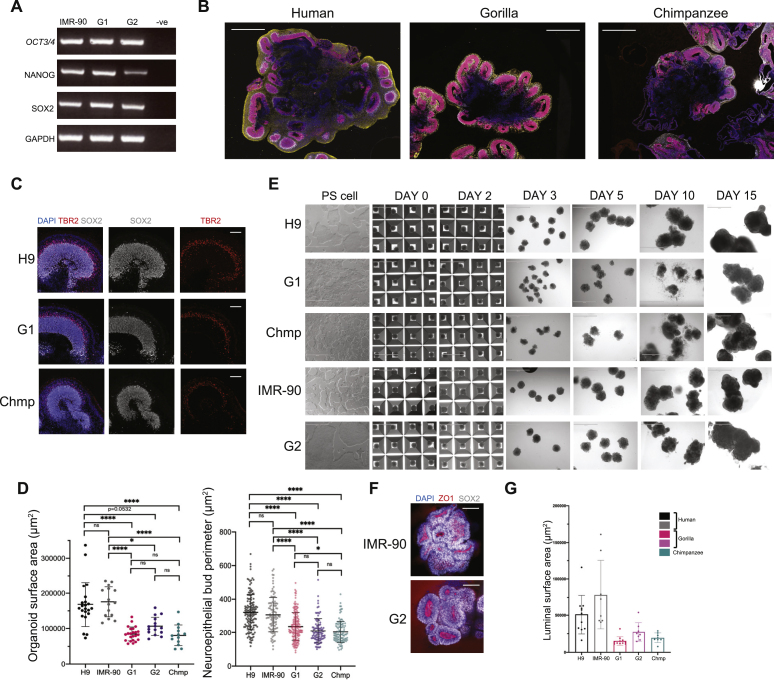
Human and ape stem cells and organoids are highly comparable in terms of identity and morphology, related to Figure 1 A. RT-PCR shows expression of pluripotency markers (*OCT3/4*, *NANOG*, *SOX2*) and *GAPDH* loading control in IMR-90, G1 and G2 cell lines. –ve is the water negative control. B. 5-week organoids stained for neural progenitor marker SOX2 (red), dorsal telencephalic/intermediate progenitor marker TBR2 (gray), neuronal markers TUJ1 (human) and HuCD (gorilla, chimpanzee) in yellow, and DAPI (blue) showing human (H9) derived organoids become larger in overall size than gorilla (G1) and chimpanzee (Chmp) organoids. Scale bar: 1mm. C. Immunofluorescent staining of representative 5-week ape organoids for neural progenitor marker SOX2, intermediate progenitor marker TBR2 and DAPI show cortical lobules of similar thickness and relative proportions of cell types. Scale bar: 100 μm. D. Quantification of the area occupied by individual organoids (left) and the perimeter of visible neuroepithelial buds (right) from brightfield images of day 10 organoids generated from all cell lines tested: human (H9, IMR-90), chimpanzee (Chmp), and gorilla (G1, G2), revealing human organoids grow consistently larger than nonhuman ape organoids. Mean organoid area: IMR-90 = 175,708 μm^2^, G2 = 106,589 μm^2^; H9, G1, Chmp as reported in Figure 1C. Mean neuroepithelial bud perimeter: IMR-90 = 307 μm, G2 = 211 μm; H9, G1, Chmp as reported in Figure 1C. ^∗^p < 0.05, ^∗∗∗∗^p < 0.0001, Kruskal-Wallis and post hoc Dunn’s multiple comparisons test, n (IMR-90) = 15 organoids and 105 neuroepithelial buds from 5 independent batches, n (G2) = 15 organoids and 94 neuroepithelial buds from 5 independent batches, n (H9, G1 and Chmp) = reported in Figure 1C. E. Representative brightfield images of pluripotent stem cells and organoids taken throughout the differentiation protocol. Note the more rounded neuroepithelium observed in day 5 organoids generated from nonhuman ape (Chmp, G1 and G2) cells versus day 5 organoids generated from human (H9, IMR-90) cells. Scale bar: 1 mm. F. Representative immunofluorescence images of the center of whole mount human (IMR-90) and gorilla (G2) day 5 organoids with staining for ZO1 and SOX2 showing polarized neural progenitor cells organized around rounded (gorilla) and more convoluted (human) ZO1 positive apical lumens. Scale bar: 100 μm. G. Quantification of the surface area of the largest apical lumen per day 5 organoid showing calculations for organoids derived from all cell lines tested. The data reveals the same trend of more expanded buds in human versus non-human ape. Mean luminal surface area: IMR-90 = 78,463 μm^2^; G2 = 27,426 μm^2^; H9, G1, Chmp as reported in Figure 1F.

**Figure 1:**
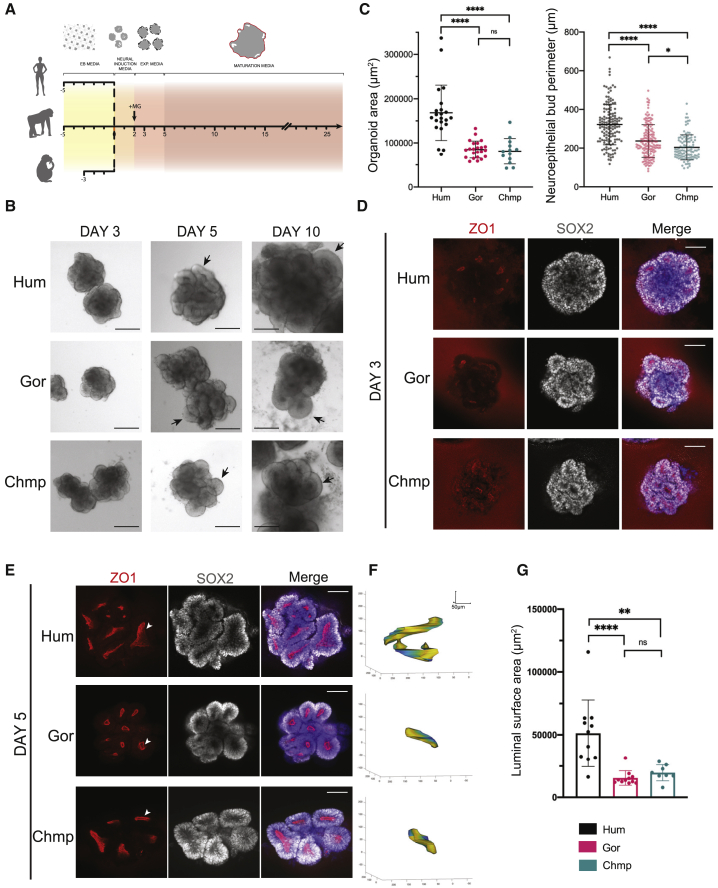
Human telencephalic organoids are larger with extended apical lumens (A) Schematic of the timeline for generating brain organoids from human, gorilla, and chimpanzee stem cells. Colors represent changes in media and stages of the protocol. +MG represents Matrigel embedding. Note chimpanzee organoids have an EB stage that is 2 days shorter than human and gorilla. (B) Bright field images of human (H9, top panels), gorilla (G1, middle panels), and chimpanzee (Chmp, bottom panels) organoids at days 3, 5, and 10. Black arrows indicate neuroepithelial buds, which appear more elongated in the human beginning at day 5. Scale bar, 200 μm. (C) Quantification of bright field images at day 10 show human (H9) neural tissue is enlarged relative to gorilla (G1) and chimpanzee (Chmp). Left: area occupied by individual organoids. Right: visible perimeter of individual neuroepithelial buds. Mean organoid area: H9 = 168,327 μm^2^; G1 = 84,876 μm^2^; Chmp = 81,086 μm^2^. Mean neuroepithelial bud perimeter: H9 = 322 μm; G1 = 237 μm; Chmp = 204 μm. ^∗^p < 0.05, ^∗∗∗∗^p < 0.0001, Kruskal-Wallis and post hoc Dunn’s multiple comparisons test, n (H9) = 22 organoids and 144 neuroepithelial buds from 6 independent batches, n (G1) = 23 organoids and 148 neuroepithelial buds from 5 independent batches, n (Chmp) = 13 organoids and 107 neuroepithelial buds from 3 independent batches, error bars are SD. (D and E) Representative immunofluorescence images of the center of whole mount human (H9), gorilla (G1), and chimpanzee (Chmp) organoids with staining for apical marker ZO1 and neural progenitor marker SOX2 at day 3 (D) and day 5 (E). Note the appearance of less rounded ZO1 positive apical lumens (arrowheads) in human organoids relative to nonhuman ape organoids at day 5. DAPI is in blue. Red background signal outside the organoid comes from nonspecific uneven staining of surrounding Matrigel. Scale bar, 100 μm. (F) 3D MATLAB reconstructions of apical lumens of day 5 organoids showing a representative example used for quantifications in (G). Values on the axes are in μm. Luminal surface area of reconstructed examples: human (H9) = 63,394 μm^2^; gorilla (G1) = 15,146 μm^2^; chimpanzee (Chmp) = 19,730 μm^2^. (G) Quantification of the surface area of the largest apical lumen per day 5 organoid reveals significantly expanded luminal surface areas in human versus nonhuman apes. Mean luminal surface area: human (H9) = 51,243 μm^2^; gorilla (G1) = 15,437 μm^2^; chimpanzee (Chmp) = 19,632 μm^2^. ^∗^p < 0.05, ^∗∗^p = 0.0012, ^∗∗∗∗^p < 0.0001, one-way ANOVA and post hoc Tukey’s multiple comparisons test, n (H9 and G1) = 11 organoids from 5 independent batches, n (Chmp) = 8 organoids from 2 independent batches, error bars are SD. See also Figure S1.

To investigate evolutionary differences in brain development between apes, we generated cerebral organoids from human, gorilla, and chimpanzee cell lines, including a new gorilla cell line (Figure S1A). We optimized the protocol so that an identical method could be used for all species (Figure 1A) resulting in highly comparable telencephalic tissues (Figure S1B). This was vital to be able to attribute any differences to species-specific divergence and not protocol variations. We did, however, notice that prior to neural induction, chimpanzee embryoid bodies (EBs) progressed faster than human and gorilla EBs, potentially reflecting the shorter gestational period of chimpanzees (Ardito, 1976). However, the difference in timing may also be a result of cell line differences and further study is needed. Nonetheless, in order to obtain comparable tissues of the proper identity (Figure S1C), timing was optimized and chimpanzee organoids were transferred to neural induction 2 days earlier (Figure 1A). All steps subsequent to germ layer determination were identical across the species.

Human organoids were consistently larger than gorilla and chimpanzee organoids at a stage when telencephalic neuroepithelial buds had formed and were undergoing expansion (Figures 1B, 1C, and S1D). We examined several time points during neuroepithelial expansion (Figure S1E) revealing similar size (Figure 1B) and tissue architecture (Figure 1D) across species at 3 days after neural induction. At day 5, however, gorilla and chimpanzee organoids presented more rounded circular neuroepithelial buds, while human organoids showed more elongated neuroepithelial buds (Figure 1B). Staining for ZO1, a tight junction protein that marks the apical surface of polarized epithelia (Ando-Akatsuka et al., 1999; Medelnik et al., 2018), revealed smaller, more rounded apical lumens in nonhuman ape organoids (Figures 1E and S1F). Using a custom image analysis pipeline, apical lumens were segmented and reconstructed in 3D, revealing species-specific differences in luminal shape and size (Figure 1F). Quantification of the largest lumen per organoid revealed that human organoids were capable of forming larger lumens than those of the other apes (Figures 1G and S1G).

**Figure S2:**
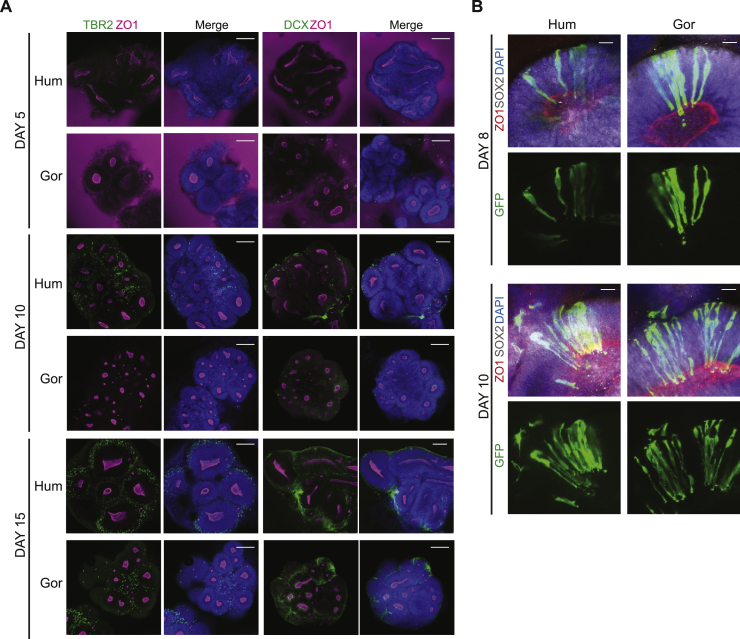
Ape NE cells undergo cell shape transition before the onset of neurogenesis, related to Figure 2 A. Representative immunofluorescence images through the center of whole mount organoids derived from human (H9) and gorilla (G1) at days 5, 10 and 15 post-neural induction with staining for intermediate progenitor cells (TBR2), newborn neurons (DCX), ZO1 and DAPI, showing that neurogenesis has not started by day 5, is commencing at day 10 and is underway by day 15. Scale bar: 100 μm. B. Representative immunofluorescence images through whole mount organoids showing the morphology of neural progenitor cells revealed by sparse labeling with viral GFP with staining for ZO1, SOX2 and DAPI for human (H9) and gorilla (G1) organoids at days 8 and 10. Note in both species the thin, elongated tNE cell morphologies on day 8 and RG cell morphologies on day 10 with stereotypical elongated narrowed apical and basal processes. Scale bar: 20 μm.

The most likely explanation for expanded lumens would be a delay in the switch from symmetrically expanding NE cells to neurogenic RG cells. We therefore tested whether at this stage ape organoids had already switched to generating neurons by staining for neurogenic TBR2^+^ intermediate progenitors and DCX^+^ newborn neurons. In human and gorilla organoids, neither cell type was present at day 5, only becoming clearly evident between days 10 and 15 (Figure S2A). This suggests that a faster neurogenic switch in ape compared to human does not explain the difference in size of cortical tissues.

### The NE to RG switch involves a transitioning NE cell morphotype that is delayed in human

**Figure 2:**
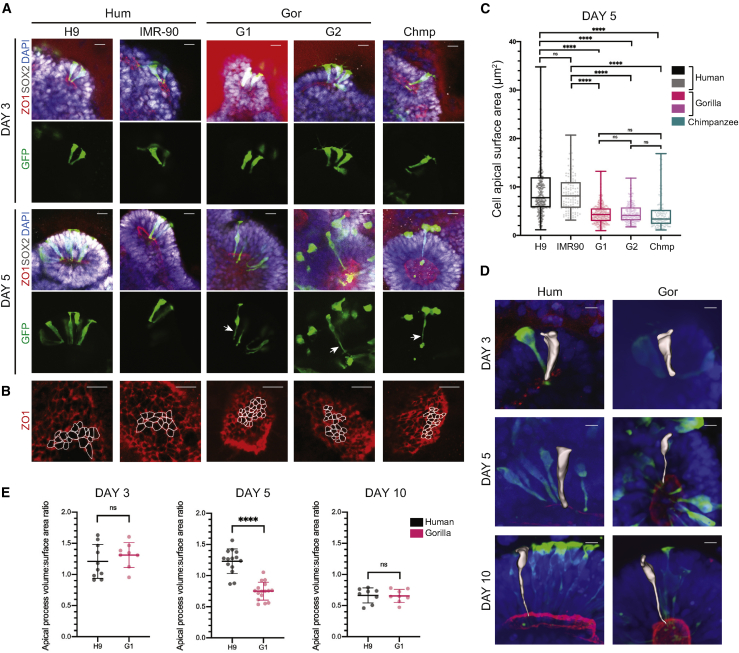
Human NE cells exhibit species-specific differences in cell shape (A) Representative immunofluorescence images through whole mount organoids showing the morphology of neural progenitor cells (SOX2^+^), polarized around apical (ZO1^+^) lumens, revealed by sparse labeling with viral GFP in human (H9, IMR-90), gorilla (G1, G2), and chimpanzee (Chmp) organoids. Day 3 cells (top panels) are columnar and exhibit typical NE morphology. Day 5 (bottom panels) human cells still appear columnar, whereas gorilla and chimpanzee cells show a thinning of apical processes (arrows). DAPI is in blue. Red background signal outside the organoid comes from nonspecific uneven staining of surrounding Matrigel. Scale bar, 20 μm. (B) Immunofluorescent staining for ZO1 on the surface of apical lumens showing the apical surface areas of individual progenitor cells at day 5 in human (H9, IMR-90), gorilla (G1, G2), and chimpanzee (Chmp) organoids. Perimeters of some individual progenitor cells are delineated in white. Scale bar, 10 μm. (C) Quantification of the apical surface area of individual neural progenitor cells of day 5 organoids show significantly smaller apical surface sizes of gorilla (G1, G2) and chimpanzee (Chmp) progenitor cells compared to human (H9, IMR-90). Measurements were performed on delineated ZO1 cell perimeters as shown in (B). Mean apical surface area/cell: H9 = 9.39 μm^2^, IMR-90 = 8.53 μm^2^, G1 = 4.48 μm^2^, G2 = 4.54 μm^2^, and Chmp = 3.55 μm^2^. ^∗∗∗∗^p < 0.0001, Kruskal-Wallis and post hoc Dunn’s multiple comparisons test, n (H9) = 341 cells from 8 organoids from 2 independent batches, n (IMR-90) = 121 cells from 4 organoids of 1 batch, n (G1) = 321 cells from 9 organoids from 2 independent batches, n (G2) = 172 cells from 5 organoids of 1 batch, and n (Chmp) = 277 cells from 6 organoids and 2 independent batches. Boxplots show median with whiskers representing min-max values; data points on the boxplots represent individual cells. (D) Representative immunofluorescence images through whole mount human (H9) and gorilla (G1) organoids with superimposed individual segmented cells (white) showing the 3D morphology of individual GFP^+^ progenitors. Note the thinning of apical processes observed in gorilla at day 5 that becomes pronounced in both species by day 10. Scale bar, 10 μm. (E) Quantification of the volume normalized to surface area of the apical processes of human (H9) and gorilla (G1) neural progenitor cells on day 3 (left), day 5 (middle), and day 10 (right), showing significantly reduced apical volumes in gorilla cells relative to human at day 5. The apical processes of segmented cells directly below the cell body were used for quantification. Mean apical process volume:surface area ratio: human day 3 = 1.21, gorilla day 3 = 1.31, human day 5 = 1.23, gorilla day 5 = 0.76, human day 10 = 0.66, and gorilla day 10 = 0.65. Mann-Whitney U, ^∗∗∗∗^p < 0.0001, two-tailed, n (day 3 human) = 10 cells, n (day 3 gorilla) = 8 cells, n (day 5 human) = 14 cells, n (day 5 gorilla) = 16 cells, n (day 10 human) = 8 cells, and n (day 10 gorilla) = 8 cells. Error bars are SD. See also Figure S2.

Another possible explanation for the difference in tissue morphology is a difference in cell morphology. Sparse viral labeling with GFP revealed progenitors with a wide, columnar shape characteristic of NE cells at day 3 (Figure 2A). At day 5, however, when the difference in tissue architecture first appears, human NE cells were still wide and columnar in shape, whereas chimpanzee and gorilla NE cells exhibited more constricted apical processes (Figure 2A). To quantify these differences, we measured the apical surface area of individual progenitor cells using ZO1 staining to delineate apical edges at the lumen (Figure 2B). At day 5, the apical surface of nonhuman ape NE cells was significantly more constricted compared to human (Figure 2C). These findings point to species-specific differences in cell shape and reveal an apically constricted transition state, herein referred to as transitioning NE (tNE), that occurs prior to the change in cellular identity and neurogenesis characteristic of RG cells.

To further quantify cell shape, we turned to higher resolution spinning-disk confocal imaging with segmentation of individual cells. Because our findings were consistent within species and across nonhuman ape cell lines, we focused on a comparison of organoids from one representative human and one representative ape cell line. Although NE cells of the two species exhibited similar morphologies at day 3, gorilla NE cells at day 5 exhibited decreased apical process volume compared with human as a result of their highly constricted morphology (Figures 2D and 2E). Human cells instead exhibited this more elongated, narrow shape at day 8 (Figure S2B), whereas by day 10, both human and gorilla NE cells exhibited a similarly elongated and constricted morphology typical of RG cells (Figures 2D, 2E, and S2B).

**Figure 3:**
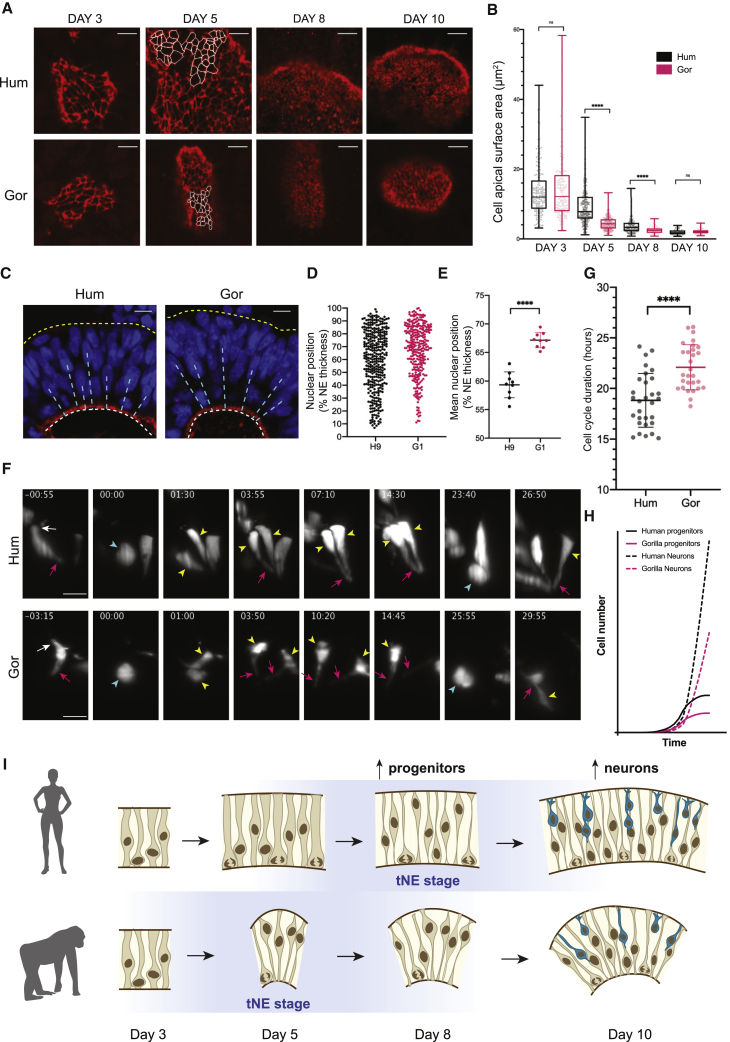
Delayed human NE transition is associated with a shorter cell cycle (A) Immunofluorescent staining for ZO1 on the surface of apical lumens of human (H9) and gorilla (G1) organoids revealing apical surface areas of individual neural progenitor cells at days 3, 5, 8, and 10. Perimeters of some individual cells of day 5 organoids are delineated in white. Scale bar, 10 μm. (B) Quantification of the surface area of individual human (H9) and gorilla (G1) NPCs between day 3 and 10 showing a gradual reduction in apical surface area over time in both species by 7-fold. Note gorilla cells are more constricted than human during the transitioning period from days 5 to 8. Measurements were performed on delineated ZO1 cell perimeters as demonstrated in (A). Mean apical surface area/cell: human day 3 = 13.82 μm^2^, gorilla day 3 = 14.80 μm^2^, human day 8 = 3.86 μm^2^, gorilla day 8 = 2.46 μm^2^, human day 10 = 1.92 μm^2^, gorilla day 10 = 2.13 μm^2^, and human and gorilla day 5 are reported in Figure 2C. ^∗∗∗∗^p < 0.0001, per time point Mann-Whitney U, two-tailed, n (day 3 human) = 164 cells from 8 organoids and 2 batches, n (day 3 gorilla) = 176 cells from 8 organoids and 2 batches, n (day 8 human) = 171 cells from 4 organoids of 1 batch, n (day 8 gorilla) = 55 cells from 2 organoids of 1 batch, n (day 10 human) = 68 cells from 3 organoids of 1 batch, n (day 10 gorilla) = 74 cells from 4 organoids of 1 batch, and n (day 5) = reported in Figure 2C. (C) Representative immunofluorescence images showing the position of nuclei (DAPI, blue) of neural progenitor cells relative to the apical surface (ZO1, red) in neuroepithelial buds of day 5 human (H9) and gorilla (G1) organoids. Dashed lines in white and yellow represent apical and basal surfaces of the neuroepithelial bud respectively. Some nuclear distances from the apical surface are shown as dashed lines in cyan, and as quantified in (D) and (E). Note the more basal distribution of nuclei in gorilla tissue. Scale bar, 10 μm. (D and E) Quantification of the position of nuclei of human (H9) and gorilla (G1) neural progenitor cells relative to the apical surface as a percentage of apicobasal neuroepithelial (NE) thickness. Measurements were performed on images as shown in (C) (D) Quantification of positions of individual cells showing gorilla progenitors located more basally. (E) Mean nuclear position per neuroepithelial bud showing gorilla cells located significantly more basal. Mean nuclear position: human = 59.34%, gorilla = 67.14%. ^∗∗∗∗^p < 0.0001, Student’s t test, unpaired, two-tailed, n (human) = 335 nuclei from 9 organoids and 3 independent batches, and n (gorilla) = 307 nuclei from 9 organoids and 3 independent batches. Error bars are SD. (F) Still frames of live imaging of neural progenitor cells sparsely labeled with GFP (grayscale) in human (H9) (Video S2) and gorilla (G1) (Video S3) organoids covering an entire cell cycle between day 3 and 4.5. Note the presence of neural progenitor cells with columnar NE morphology in both species prior to the first division (00:00, reference time point) and loss of basal process (white arrowheads) during cell mitosis (blue arrowheads). Note daughter cells (yellow arrowheads) show thicker apical processes (magenta arrowheads) in human relative to the more constricted morphology of gorilla apical processes. Time shown in hours:minutes. (G) Quantification of cell cycle duration of human (H9) and gorilla (G1) neural progenitor cells imaged between days 3 and 5 showing significantly longer cell cycles in gorilla. Mean cell cycle length: human = 18.83 h, gorilla = 22.10. ^∗∗∗∗^p < 0.0001, Mann-Whitney U, two-tailed, n (human) = 30 cells from 5 independent batches, n (gorilla) = 29 cells from 5 independent batches. Error bars are SD. (H) Growth curve modeling of the predicted effect that differences in cell cycle length, as measured in (G), would have on human and gorilla progenitor numbers (bold lines) and on neuron numbers (dashed lines) with a 1.9-fold expected increase in human for both. (I) Schematic summarizing the morphological changes in neural progenitor cells. Progenitor cells of apes undergo a gradual transition from NE to tNE to neurogenic RG cells. Nonhuman ape cells show tNE morphologies (blue background) earlier than human cells, which show shorter cell cycles leading to increased neuron numbers. See also Figure S3 and Videos S1, S4, and S5.

We next performed a temporal assessment of progenitor cell apical surface area as labeled by ZO1. At day 3, the average apical surface of cells was large and indistinguishable between human and gorilla. By day 10, both human and gorilla exhibited complete transition with apical surface area decreasing by 7-fold (Figures 3A and 3B). However, the transition was delayed in human, where apical surface area at day 8 more closely matched that of gorilla at day 5. These findings reveal a later switch to apically constricted tNE morphologies in human compared with nonhuman ape organoids.

**Figure S3:**
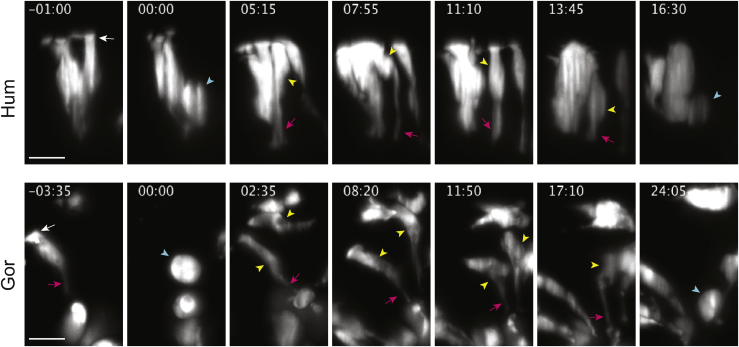
Live imaging of human and gorilla cerebral organoids, related to Figure 3 Representative still frames of live imaging of neural progenitor cells sparsely labeled with GFP (greyscale) in human (H9) (Video S4) and gorilla (G1) (Video S5) organoids covering an entire cell cycle between day 4 and 5, showing a significantly longer cell cycle in gorilla, calculated as the period between cell divisions (blue arrowheads). Parent and daughter cells (yellow arrowheads) show more constricted apical processes (magenta arrowheads) in gorilla relative to the apical process of human progenitors which thickens into a NE morphology in the frames following the first division. Note the loss of the basal process (white arrowhead) during mitosis. Time shown in hours:minutes, 00:00 marks the first division and reference time point

Cell shape is closely linked to cell cycle through interkinetic nuclear migration, a behavior characteristic of NE and RG cells in which the nucleus moves along the apicobasal axis in time with the cell cycle. We investigated nuclear positioning and observed more frequent apical nuclei in human NE at day 5 (Figures 3C–3E), suggesting a difference in interkinetic nuclear migration and pointing to potential cell cycle differences. To explore this possibility, we performed live imaging of whole organoids over the course of several days, using SiMView adaptive light sheet microscopy (Video S1) (McDole et al., 2018). As previously described (Subramanian et al., 2017), we observed a transient loss of the basal process during NE cell division in both human and gorilla. Following the division, the apical processes of human NE daughter cells appeared thicker than those of gorilla (Figures 3F and S3; Videos S2, S3, S4, and S5), consistent with our findings above. We also measured cell cycle lengths, revealing a shorter cell cycle in human NE cells (Figure 3G). Mathematical growth curve modeling of the effect of this 3-h cell cycle difference predicted a progenitor and neuron number increase in human of 1.9-fold (Figure 3H). These findings together suggest changes in cell shape are coupled to the cell cycle, and a delay in this transition in human leads to a larger founder progenitor pool and therefore an expected increase in neuron number (Figure 3I).

Video S1. Side-by-side movie of SiMView imaged human and gorilla organoids, related to Figure 3 Live imaging using SiMView adaptive light sheet microscopy of progenitor cells sparsely labeled with GFP in a human cerebral organoid (left) and a gorilla cerebral organoid (right) beginning on day 3. Images were acquired every 5 minutes. Note the appearance of labeled radially organized neuroepithelial cells beginning at approximately 6 hours of imaging and the progressive thinning of the apical processes, particularly in gorilla which becomes more apparently different at approximately 40 hours. Time shown in day:hours:minutes. Scale bar: 25 μm.

Video S2. Human cerebral organoid movie beginning on day 3, related to Figure 3 Live imaging using SiMView of progenitor cells sparsely labeled with GFP in a human cerebral organoid beginning on day 3. Images were acquired every 5 minutes. Time shown in hours:minutes. Scale bar: 20 μm.

Video S3. Gorilla cerebral organoid movie beginning on day 3, related to Figure 3 Live imaging using SiMView of progenitor cells sparsely labeled with GFP in a gorilla cerebral organoid beginning on day 3. Images were acquired every 5 minutes. Time shown in hours:minutes. Scale bar: 20 μm.

Video S4. Human cerebral organoid movie beginning on day 4, related to Figure 3 Live imaging using SiMView of progenitor cells sparsely labeled with GFP in a human cerebral organoid beginning on day 4. Images were acquired every 5 minutes. Time shown in hours:minutes. Scale bar: 20 μm.

Video S5. Gorilla cerebral organoid movie beginning on day 4, related to Figure 3 Live imaging using SiMView of progenitor cells sparsely labeled with GFP in a gorilla cerebral organoid beginning on day 4. Images were acquired every 5 minutes. Time shown in hours:minutes. Scale bar: 20 μm.

### RNA sequencing analysis captures dynamics of gene expression across multiple early time points

**Figure 4:**
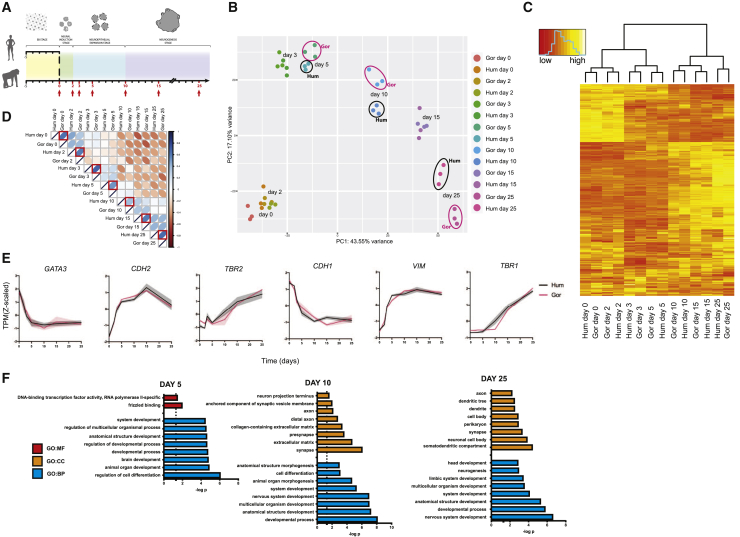
Ape organoids exhibit comparable developmental molecular trajectories (A) Schematic of the timeline for human and gorilla brain organoids with RNA-seq collection time points shown in red. 3 biological replicates of organoids derived from human (H9) and gorilla (G1) cells were collected at each of the 7 time points. (B) PCA biplot of PC1 versus PC2 performed on *Z*-scaled log2-transformed TPMs of the 3,000 most variable genes. Samples are color-coded by time point and species. Note samples separating primarily by time point with a slight separation between samples of different species at days 5, 10, and 25, highlighted by ellipses (black ellipses for human and fuchsia for gorilla). (C) Heatmap with hierarchical clustering based on shared expression pattern (*Z* scores of log2-transformed TPMs of the 3,000 most variable genes) between samples. The dendrogram shows samples clustering by time point. (D) Pearson’s correlation map using *Z*-scaled log2-transformed TPMs of all genes. Darker blue depicts stronger positive correlation between samples and darker red a stronger negative correlation. Red boxes highlight the correlation between species at matched time points. Note lower correlation between species at days 5 and 10. Pearson’s correlation coefficient: r = 0.66 (day 0), 0.63 (day 2), 0.66 (day 3), 0.52 (day 5), 0.40 (day 10), 0.62 (day 15), and 0.66 (day 25). (E) Temporal expression pattern (*Z*-scaled) of characteristic developmental markers show predicted expression dynamics. Non-neural ectoderm markers (*GATA3*, *CDH1*) are lost rapidly, followed by a gain in neural progenitor markers (*CDH2*, *VIM*), and a later increase in intermediate progenitor (*TBR2*) and early-born neuron (*TBR1*) markers. Shaded error bar is SD. (F) GO term enrichment analysis on the top 300 genes driving species variance at days 5, 10, and 25. Shown are the 8 most significant (p < 0.05) enrichments for GO categories molecular function (GO:MF), cellular compartment (GO:CC), and biological process (GO:BP). See also Figure S4 and Data S1.

**Figure S4:**
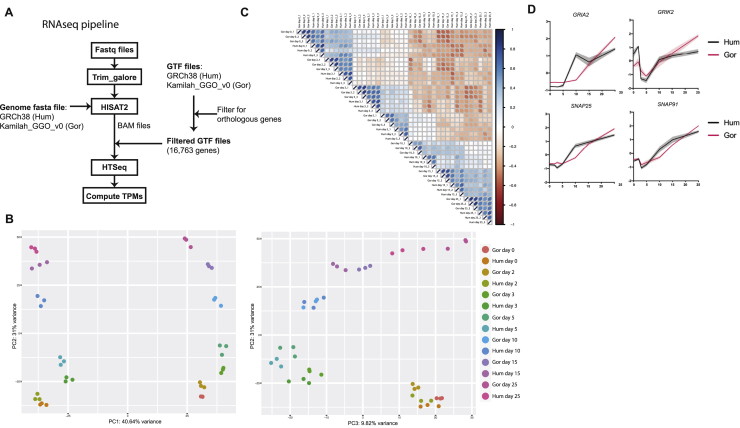
RNA-seq data analysis pipeline and normalization, related to Figure 4 A. Workflow summarizing the RNaseq analysis pipeline (see methods). B. PCA performed using log2-transformed TPMs. Samples are color-coded by time point and species. Graph of PC1 versus PC2 (left) shows biological replicates grouping together, samples separating by species along PC1 and separating by time point along PC2. Plotting PC2 versus PC3 (right) shows samples separating by time point and not species. C. Pearson’s correlation of all samples using z-scaled log2-transformed TPMs of all genes. Darker blue depicts stronger positive correlation between samples and darker red a stronger negative correlation. Data shows the strongest correlation between biological replicates within a species followed by between species time point-matched samples. Correlation between species is lowest at day 5 and 10. D. Temporal expression pattern (z-scaled) of genes related to synaptic formation and maturation (*GRIA2*, *GRIK2*, *SNAP25*, *SNAP91*). Shaded error bar is SD.

To identify what factors might be controlling these changes in cell shape, we examined the transcriptome profile of human and gorilla organoids. Although there are a number of published cerebral organoid single-cell RNA sequencing (RNA-seq) datasets, including from human and chimpanzee (Kanton et al., 2019; Mora-Bermúdez et al., 2016; Pollen et al., 2019), those collected to date do not capture early NE to RG transition stages with a high enough temporal resolution. Therefore, we decided to perform bulk RNA-seq of a large number of organoids across many early time points and replicates to identify high confidence differentially expressed genes. In total, we performed RNA-seq of 42 samples of ∼9,000 organoids, collected from day 0, corresponding to pre-neurulation tissue, up until day 25, corresponding to fully committed neurogenesis (Figure 4A). To compare expression of genes shared between both species, transcripts per millions (TPMs) were calculated using a filtered list of annotated genes for which an orthologous gene was present in both species (Figure S4A). Principal component analysis (PCA) of the samples showed biological replicates grouping together, highlighting the robustness and reproducibility of the protocol (Figure S4B). It also showed samples separated by species along PC1, followed by grouping by time along PC2 and PC3. PC1 was likely driven by technical artifacts arising from read mapping and annotation differences coming from the reference genomes of the two species. Therefore, to normalize within each species and at the same time focus on dynamics rather than absolute levels, the expression of each gene was normalized by *Z*-scaling across time.

PCA of normalized data showed samples of the same time point across species grouped together (Figure 4B). Hierarchical clustering on mean *Z* scores (Figure 4C) and Pearson’s correlation analysis (Figures 4D and S4C) also showed grouping by time rather than species. A panel of genes with characteristic developmental roles showed expected patterns of expression in both species (Figure 4E). These data further confirm the proper identity and developmental trajectory of organoids from both species and demonstrate they are highly comparable.

Although organoids collected at the same time point appeared highly correlated across species, the PCA biplot revealed a degree of separation between species at days 5, 10, and 25 (Figure 4B). We performed Gene Ontology (GO) term enrichment analysis on the top 300 genes driving the variance at these time points (Data S1). GO terms at all time points were generally associated with nervous system development, with earlier day 5 time point terms primarily covering organ development and morphogenesis, and day 25 terms associated with neuronal compartments such as dendrites and synapses, as well as neurogenesis (Figure 4F). We further examined some of the genes driving the variance at day 25 and saw a steeper rate of increase in genes associated with synaptic formation and maturation in gorilla (Figure S4D). This is consistent with previous studies (Kanton et al., 2019; Liu et al., 2012; Marchetto et al., 2019) suggesting that human neurons mature at a slower rate.

### Species-specific differences in gene expression and biological processes related to cell morphogenesis during NE to RG transition

**Figure 5:**
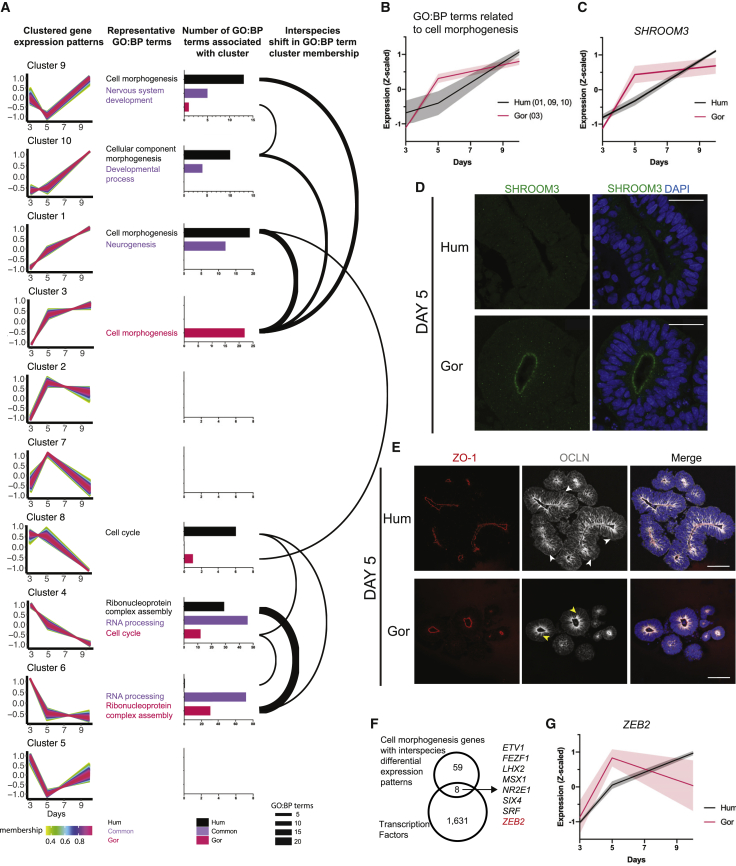
The human neuroepithelium exhibits differential temporal dynamics of morphogenesis genes (A) Clustering genes by temporal expression dynamics shows species differences in GO:BP term enrichment. Columns from left to right: far left, TCseq clusters with genes in each cluster plotted with their temporal expression (z-scaled) and color-coded by membership value (degree to which data points of a gene belong to the cluster, pink represents high membership values). The 10 clusters are ordered (top to bottom) based on similarity in expression pattern. Middle left: representative GO:BP term from shared (purple), human-exclusive (black), or gorilla-exclusive (fuchsia) terms for each cluster. Middle right: histograms show the number of enriched (p < 0.05) GO:BP terms found in both species (purple), exclusively in human (black) or gorilla (fuchsia) per cluster. Axis range: 0–8 (cluster 2,5,7,8); 0–15 (cluster 9,10); 0–20 (cluster 1); 0–25 (cluster 3); 0–50 (cluster 4); 0–80 (cluster 6). Far right: weighted arc network graph visualizing interspecies differences in the enrichment/membership of specific GO:BP terms per cluster. The bases of the arc are aligned to both a human (black) and a gorilla (fuchsia) bar from the histogram in the adjacent panel, highlighting the species-specific shifts in expression patterns associated with specific GO:BP terms. Weight/thickness of the arc is dictated by the number of GO:BP terms enriched in a species-exclusive manner “moving” between clusters in the defined pattern. (B) Mean temporal expression pattern (z-scaled) of genes in clusters enriched for “cell morphogenesis”-related GO:BP terms (human clusters 1, 9, 10, and gorilla cluster 3). (C) Temporal expression pattern (*Z*-scaled) of *SHROOM3*, a gene involved in cell morphogenesis and apical constriction. (D) Immunofluorescent staining of day 5 organoids for SHROOM3 showing strong apical expression in gorilla (G1) neuroepithelium, but not human (H9) at this time point. Scale bar, 40 μm. (E) Immunofluorescent staining of day 5 organoids for OCLN showing expression spread along the apicobasal length of human (IMR-90) progenitor cells (white arrowheads) but more limited apically (yellow arrowheads) in gorilla (G1) progenitor cells. DAPI is shown in blue. Scale bar, 100 μm. (F) Venn diagram summarizing search for cell morphogenesis-related transcription factors with species-specific expression patterns. (G) Mean temporal expression pattern (*Z*-scaled) of *ZEB2*, showing peak expression earlier in gorilla (G1) than human (H9) organoids. Shaded error bars are SD. See also Figure S5 and Data S1.

Pearson’s correlation coefficient was also lower at day 5 and day 10 than any of the other time points (Figure 4D), coinciding with our observed differences in cell shape. We next focused on gene expression changes arising between day 3 and day 10, which would cover the time taken in both species for progenitors to transition from NE through tNE to RG morphologies. We analyzed abundantly expressed genes (TPM >10) with reproducible expression patterns between biological replicates (squared difference <6) and those with more dynamic expression patterns over the 3 time points (fold change >1.5 between any two time points in at least one species). This resulted in a list of 2,905 genes that were then subjected to analysis by TCseq (Wu and Gu, 2020) to cluster genes with similar temporal patterns. Focusing on 3 time points meant that temporal patterns could be represented by and separated into 10 clusters with distinct shapes (Figure 5A). During TCseq analysis, biological replicates were kept separate in order to further filter for genes with reliable cluster assignment (i.e., genes where ≥2 replicates were assigned to the same cluster), yielding a list of 2,342 genes per species (Data S1).

To assign biological functions to the identified temporal patterns, we performed GO term enrichment analysis on each TCseq cluster, keeping human and gorilla genes separate (Data S1). We focused on GO:BP terms found in both species and examined whether each term was found in the same cluster for both species, or different clusters which would indicate a species-specific change in temporal dynamics for that biological function. This revealed a number of GO:BP terms associated with clusters common to both species (Figure 5A), for example, GO:BP terms related to “neurogenesis” and “RNA processing” exhibited identical expression dynamics in both species.

**Figure S5:**
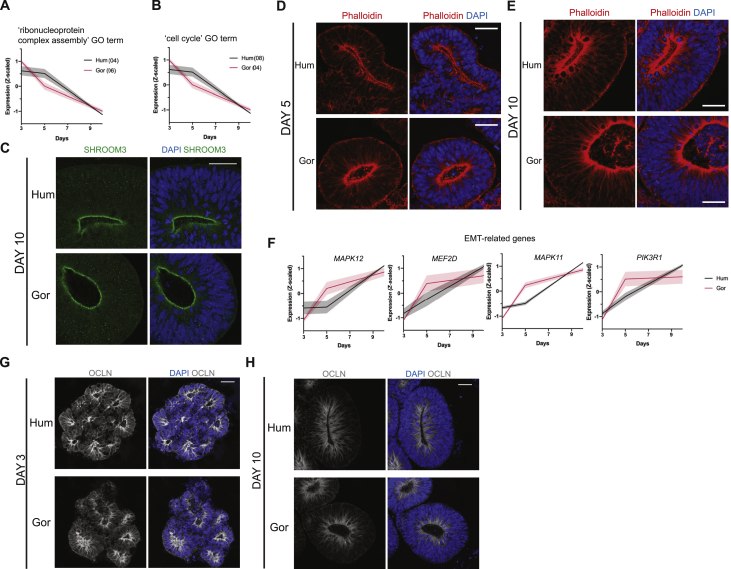
Expression patterns of key factors with differential temporal dynamics, related to Figure 5 A, B. Mean temporal expression pattern (z-scaled) of genes in clusters enriched for: A. ‘ribonucleoprotein complex assembly’-related GO:BP terms (human cluster 4, gorilla cluster 6) B. ‘cell cycle’-related GO:BP terms (human cluster 8, gorilla cluster 4). C. Immunofluorescent staining of day 10 organoids for SHROOM3 shows strong apical expression in neuroepithelium of both species (H9, G1). Scale bar: 40 μm. D, E. Immunofluorescent staining for F-actin (Phalloidin) and DAPI of human (H9) and gorilla (G1) organoids on day 5 (D) and day 10 (E) showing weaker apical accumulation in human versus gorilla at day 5 with strong apical accumulation observed in both species by day 10. Scale bar: 40 μm. F. Mean temporal expression (z-scaled) of EMT-related genes enriched for WP term ‘Epithelial to mesenchymal transition in colorectal cancer’ *MAPK12*, *MEF2D*, *PIK3R1* and *MAPK11*, robustly changing pattern between species in the same way enrichment of ‘cell morphogenesis’-related terms changes pattern between species (Figure 5B). G, H. Immunofluorescent staining of organoids (H9, G1) for OCLN at G. day 3, showing expression along the apicobasal length of progenitor cells in both species H. day 10, showing lowered expression limited apically in both species. Scale bar: 40 μm.

GO:BP terms with differential expression dynamics between the two species represented particularly interesting biological functions. To visualize the major shifts in temporal expression patterns, we generated a weighted network graph of GO:BP terms that were uniquely present for one species in a given cluster, but present in a different cluster for the other species (Figure 5A). The largest shift of GO:BP terms was between human cluster 4 and gorilla cluster 6, where terms such as “ribonucleoprotein complex assembly” were seen to move from a pattern showing a more gradual decrease in expression in human, to one dropping sharply after day 3 in gorilla (Figure S5A). These terms were linked to the umbrella term, “RNA processing,” and point to interesting changes in alternative splicing, previously highlighted as a key driver of evolutionary differences across primates (Bush et al., 2017). Another interesting shift in GO terms was “cell cycle” moving from a cluster where expression levels were similar between day 3 and 5 in human (cluster 8), to a cluster where expression levels had already started declining by day 5 in gorilla (cluster 4) (Figure S5B). This finding fits well with the observed differences in cell cycle length (Figure 3G).

The shift that we found most relevant was in GO terms such as “cell morphogenesis” and “cellular component morphogenesis,” which were present in cluster 3 of gorilla but in clusters 1, 9, and 10 of human (Figure 5A), because differences in cell morphogenesis were precisely what we had observed. These terms were associated with genes whose expression increased more rapidly in gorilla than in human (Figure 5B), matching the more rapid transition in cell shape observed in nonhuman apes. In particular, *SHROOM3* (Figure 5C) immediately stood out due to its role in actin-mediated apical constriction (Haigo et al., 2003; Hildebrand and Soriano, 1999; Lee et al., 2007). Immunostaining for SHROOM3 at day 5 confirmed the differences detected by RNA-seq, with strong SHROOM3 localization visible along the apical surface in gorilla, whereas apical surfaces of human progenitors showed minimal to no staining (Figure 5D). By day 10, strong SHROOM3 staining was seen at the apical surface in both species (Figure S5C). Phalloidin staining also revealed more extensive accumulation of actin at the apical surface of day 5 gorilla tNE cells (Figure S5D), whereas such accumulation appeared later in human (Figure S5E). These results point to SHROOM3 and actin in apical constriction in the context of NE transition.

A closer inspection of the genes contributing to the GO:BP term shift that displayed earlier onset of expression in gorilla, revealed a group of genes involved in epithelial-to-mesenchymal transition (EMT) (Figure S5F). This was particularly interesting because the process of corticogenesis, from NE cells to neurons, is characterized by a progressive and gradual loss of epithelial features, reminiscent of EMT (Singh and Solecki, 2015). One such epithelial feature that is lost is the presence of the tight junction protein OCLN (Aaku-Saraste et al., 1996). Staining for OCLN revealed a difference in its distribution over time, going from extended staining along the entire apico-basal axis of individual cells at day 3 (Figure S5G) to a limited distribution apically at day 10 (Figure S5H). This change in its distribution was delayed in human, with day 5 organoids exhibiting a similar pattern to day 3, whereas gorilla OCLN was already more apically restricted (Figure 5E).

To identify upstream regulators of this process, we queried the set of genes with robust differential temporal dynamics (Data S1) that belonged to the “cell morphogenesis” GO:BP term for genes predicted to be transcription factors, revealing a set of 8 genes (Figure 5F). Of these, *ZEB2* stood out due to its well-described role as an EMT master regulator (Peinado et al., 2007; Stemmler et al., 2019). Furthermore, several comparative genomics analyses have pulled out *ZEB2* as a human gene under selective pressure and exhibiting various human accelerated regions (HARs) (Erwin et al., 2014; Lindblad-Toh et al., 2011; Pollard et al., 2006). *ZEB2*, which showed a shift from cluster 1 in human to 2 in gorilla, therefore stood out as a potential regulator of cell shape in this context. The expression dynamics of *ZEB2* revealed an earlier peak of expression in gorilla compared with human (Figure 5G), matching the expected expression pattern of a gene that could be initiating the tNE shape transition.

### *ZEB2* is expressed during NE to RG cell transition and this process is delayed in *ZEB2* heterozygous loss-of-function organoids

**Figure 6:**
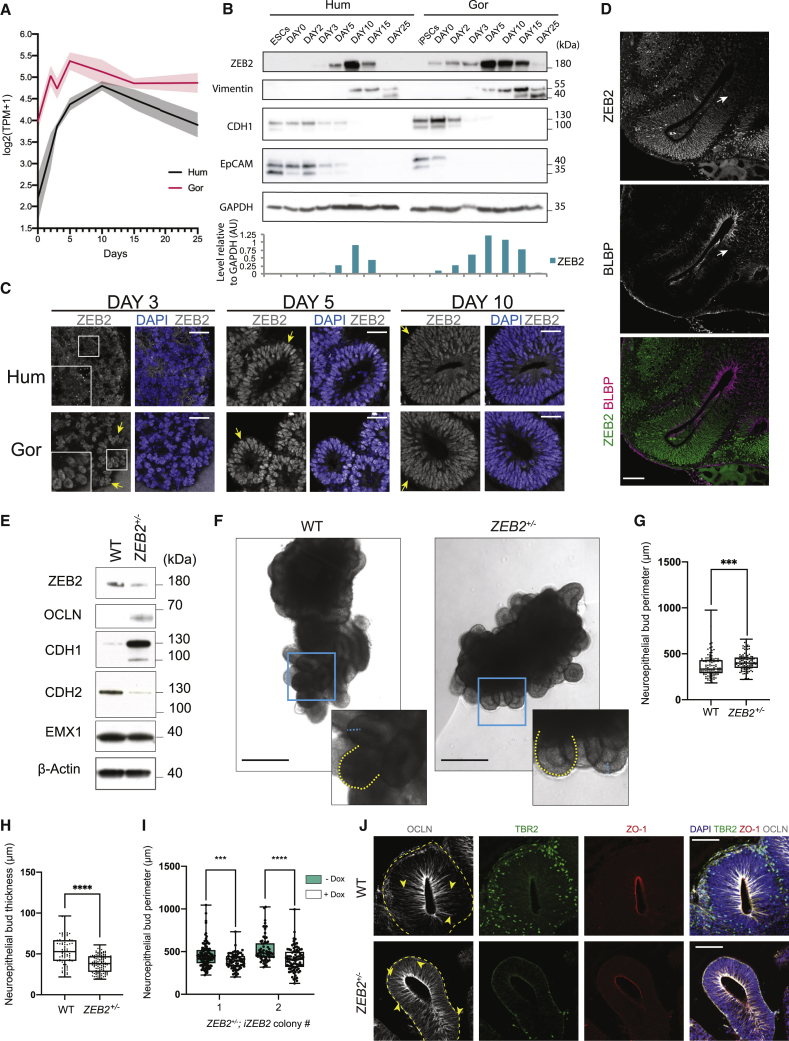
Decreased ZEB2 leads to expanded NE with delayed transition (A) Mean temporal expression pattern (log normalized transcripts per million) of *ZEB2* across the entire time series, showing peak expression earlier in gorilla (G1) than human (H9) organoids. Shaded error bars are SD. (B) Western blot expression time course from PSCs to day 25 human (H9) and gorilla (G1) organoids reveals a premature onset and higher levels of ZEB2 protein expression in gorilla compared to human. This is accompanied by a premature expression of the radial glial marker vimentin, and premature downregulation of the epithelial markers CDH1 and EpCAM in gorilla relative to human. Bottom panel shows quantification of ZEB2 relative to GAPDH (AU, arbitrary units). (C) Immunofluorescent stain for ZEB2 and DAPI in human (H9) and gorilla (G1) organoids at days 3, 5, and 10 showing neuroepithelial buds with nuclear expression (yellow arrows). Note the interspecies difference at day 3 where gorilla organoids already display nuclear expression compared to a weaker stain in most human cells. Insets show higher magnification of the boxed regions. Scale bar, 40 μm. (D) Immunofluorescence image of a day 25 human (H9) organoid showing a mutually exclusively pattern of expression between ZEB2 and the committed radial glia marker, BLBP. Scale bar, 100 μm. (E) Western blot of H9 wild-type (WT) and *ZEB2*^+/−^ organoids at day 16 for ZEB2, the tight-junction protein OCLN, the junction components CDH1 and CDH2, the dorsal telencephalic marker EMX1, and the loading control β-actin. The blots show a sizeable increase in CDH1 and OCLN and a decrease in CDH2, whereas EMX1 levels, and thus dorsal telencephalic identity, appears to be largely unaffected. (F) Representative bright field images of day 12 WT and *ZEB2*^+/−^. Insets show higher magnification of the boxed regions, dashed yellow are representative neuroepithelial bud perimeters quantified in (G), dashed turquois are representative neuroepithelial bud thicknesses quantified in (H). Scale bar, 500 μm. (G) Quantification of neuroepithelial bud perimeters of WT (n = 106) and *ZEB2*^+/−^ (n = 116) organoid buds from 27 WT and 28 *ZEB2*^+/−^ organoids at day 17, Mann-Whitney U test, two-tailed (^∗∗∗^p = 0.0001) from 3 organoid batches. (H) Quantification of neuroepithelial bud thickness of WT (n = 80) and *ZEB2*^+/−^ (n = 119) organoid buds from 26 WT and 31 *ZEB2*^+/−^ organoids at day 12, Mann-Whitney U test, two-tailed (^∗∗∗∗^p < 0.0001) from 3 organoid batches. (I) Quantification of neuroepithelial bud perimeters of two *ZEB2*^+/−^;*iZEB2* colonies treated with and without doxycycline. Colony 1: − Dox (n = 108 buds from 25 organoids), + Dox (n = 70 buds from 18 organoids). Colony 2: − Dox (n = 62 buds from 17 organoids), + Dox (n = 84 buds from 19 organoids) across 3 organoid batches. Mann-Whitney U tests, two-tailed (^∗∗∗^p = 0.0003 ^∗∗∗∗^p < 0.0001). (J) Immunofluorescence images of day 15 WT and *ZEB2*^+/−^ organoids showing increased OCLN immunostaining (yellow arrowheads) along the apico-(ZO1) basal (dashed line) axis of progenitor cells and reduced numbers of TBR2^+^ cells in *ZEB2*^+/−^ organoids compared to WT. Scale bar, 100 μm. See also Figure S6.

Further examination of the full time course of *ZEB2* expression and its overall levels in the RNA-seq data (Figure 6A) revealed *ZEB2* mRNA levels peaking at day 5 in gorilla, whereas in human *ZEB2* peaked at day 10. We next examined ZEB2 protein and its downstream targets in human and gorilla organoids by western blot, revealing an earlier onset of expression of ZEB2 in gorilla compared to human (Figure 6B). Consistent with a precocious EMT-like transition in gorilla, the epithelial cell adhesion proteins CDH1 and EpCAM showed earlier downregulation, while Vimentin, a marker of both RG and EMT, showed earlier upregulation in gorilla compared to human (Figure 6B).

**Figure S6:**
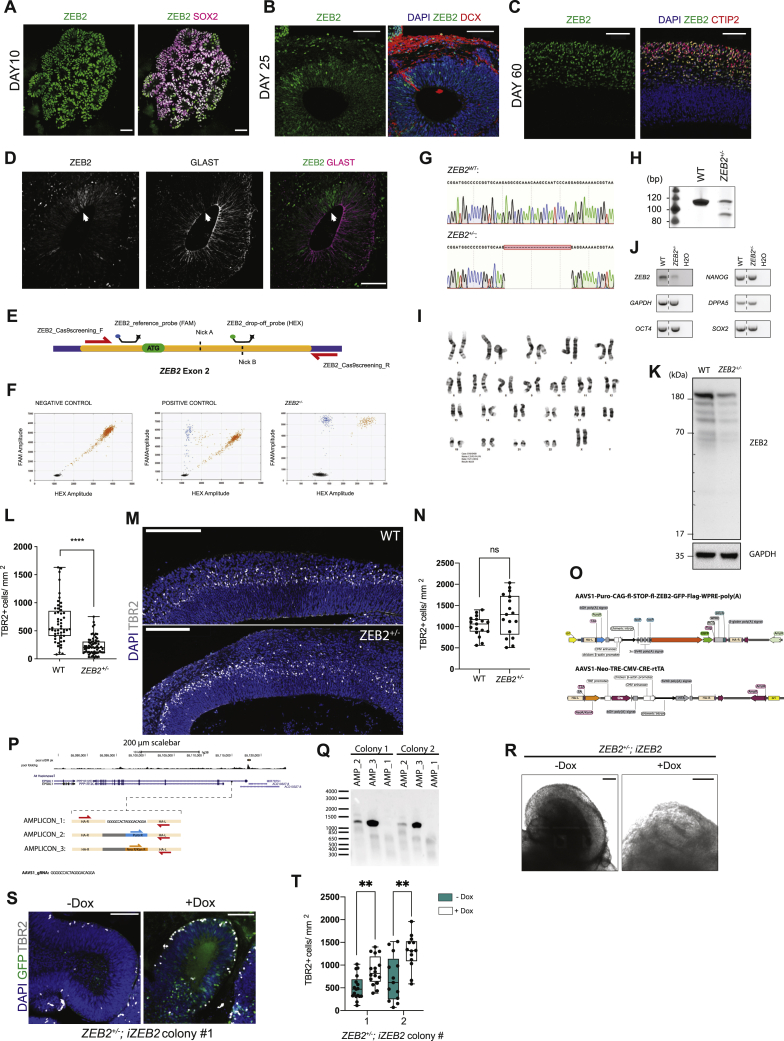
ZEB2 expression and targeting for loss of function, related to Figure 6 A. Representative immunofluorescence image showing ZEB2 expression in SOX2+ progenitor cells in day 10 human (H9) organoid. Scale bar: 50 μm. B. Representative immunofluorescence image showing a salt-and-pepper pattern of ZEB2 expression in the ventricular zone at day 25, after the onset of neurogenesis in human (H9) organoid. DCX (Doublecortin) stains newly born neurons. Scale bar: 100 μm. C. Representative immunofluorescence image of a mature day 60 human (H9) organoid revealing ZEB2 expression in CTIP2+ neurons and absence of ZEB2 staining in the ventricular zone. Scale bar: 100 μm. D. Representative immunofluorescence image of a day 25 human (H9) organoid showing a mutually exclusive pattern of expression between ZEB2 and the radial glia marker protein GLAST. Scale bar: 100 μm. E. Schematic representation of the CRISPR-Cas9n editing strategy, where the first coding exon of the *ZEB2* gene (exon 2, NCBI ref sequence NM_014795.4:182-323) was targeted by two nickases (dashed lines) and screening was performed by assaying the drop-off frequency of a HEX-labeled probe, binding to one of the nick sites, relative to a FAM-labeled reference probe binding away from the disrupted region. The exon is marked in orange while introns are marked in purple. F. Example ddPCR 2D scatter-plots of a negative control sample (HEK293 cells), showing only a FAM-HEX double positive (red) and an empty droplet cluster (black) and a positive control sample (HEK293 cells expressing WT Cas9 and *ZEB2* guides), showing a FAM-only cluster (blue) in the upper-left quadrant of the 2D plot corresponding to edited alleles. ddPCR 2D scatter-plot of the H9 *ZEB2*^*+/−*^ hESC edited line showing a 1:1 ratio between the WT and edited allele. (G). Representative chromatograms of the *ZEB2* alleles in the H9 *ZEB2*^*+/−*^ hESC cells. The CRISPR-Cas9 target region was PCR amplified with a high-fidelity polymerase, the PCR product was blunt-end cloned into the pJET1.2 vector and following purification, plasmids from different colonies carrying the insert were sequenced. Sequencing reveals that the edited allele harbors a 23 bp deletion. H. DNA-PAGE analysis of a short PCR amplicon spanning the CRISPR-Cas9 *ZEB2* target site in WT H9 and H9 *ZEB2*^*+/−*^ hESCs. The gel reveals the presence of two bands, corresponding to the WT and the edited allele in H9 *ZEB2*^*+/−*^ hESCs. I. Representative images of karyotype analysis on 20 G-banded metaphase spreads from the H9 *ZEB2*^*+/−*^ hESCs used to generate the stock. The cell line displays normal karyotype. J. RT-PCR analysis for expression of *ZEB2,* the key pluripotency markers *SOX2, NANOG, OCT4* and *DPPA5* and the loading control *GAPDH*. PCR shows that upon a ~50% reduction in *ZEB2* mRNA levels the mutant stem cells retain expression of pluripotency markers at comparable levels to WT H9 hESCs. WT and *ZEB2*^+/−^ were run on the same gel but not adjacent to each other, the dashed line indicates where the gel was spliced. K. Full length western blot for ZEB2 in WT and *ZEB2*^*+/−*^ organoids at day 15 – loading control was GAPDH L. Box and whiskers plot reporting the quantifications of the number of TBR2+ cells per unit area (TBR2+ cells/mm^2^) in day 16 WT and *ZEB2*^*+/−*^ organoids. Quantifications were performed by manual counting on n = 52 WT and n = 68 *ZEB2*^*+/−*^ ventricles corresponding to 12 organoids from 2 distinct batches. A two-tailed Mann-Whitney U test was used for statistical comparison (^∗∗∗∗^p < 0.0001). M. Representative immunofluorescence images of day 55 WT and *ZEB2*^*+/−*^ cerebral organoid buds used for quantifications shown in N. Scale bar: 200 μm. N. Box and whiskers plot reporting the quantifications of the number of TBR2+ cells per unit area (TBR2+ cells/mm^2^) in day 55 WT and *ZEB2*^*+/−*^ organoids. Quantifications were performed using an automated cell segmentation pipeline on n = 17 WT and n = 17 *ZEB2*^*+/−*^ organoid regions from 3 distinct batches. A two-tailed Mann-Whitney U test was used for statistical comparison (ns, p = 0.1139). O. Plasmid maps of the CRISPR homology-directed repair (HDR) templates used to target the AAVS1 safe-harbor locus in H9 hESC cells – top is the CAG-lox-STOP-lox-ZEB2-GFP-Flag inducible expression construct and bottom is the construct encoding CRE recombinase under the control of a tetracycline responsive promoter and the reverse tetracycline transactivator (rtTA) driven by the CAG promoter. P. UCSC Genome Browser view of the AAVS1 locus and CRISPR-Cas9 targeting strategy of intron 1 of *PPP1R12C*. The promoter-less splice-acceptor (SA), T2A peptide-linked “gene trap” is such that expression of the promoter-less selection cassette is driven by the endogenous *PPP1R12C* gene, thus effectively eliminating false-positive background arising from random integration. The panel reports the PCR genotyping strategy – upon successful targeting of the AAVS1 locus, while amplicon 1 is lost due to the size increase following insert integration, amplicons 2 and 3 are gained - see Figure S7A. Q. PCR gel showing successful genotyping of the two rescue clones used for the experiments shown. R. Representative brightfield images of day 15 *ZEB2*^*+/−*^*; iZEB2* cerebral organoids treated with and without doxycycline. Scale bar: 100 μm. S. Representative immunofluorescence images of *ZEB2*^*+/−*^*; iZEB2* treated with and without doxycycline stained for GFP, TBR2 and DAPI. Scale bar: 100 μm T. Box and whiskers plot reporting the quantifications done using an automated cell segmentation pipeline of the number of TBR2+ cells per unit area (TBR2+ cells/mm^2^) in day 15 *ZEB2*^*+/−*^*; iZEB2* organoids - colony 1: -Dox (n = 17 organoid regions), +Dox (n = 16 organoid regions); colony 2: -Dox (n = 13 organoid regions), +Dox (n = 13 organoid regions) from three independent batches. Mann-Whitney U tests, two-tailed (^∗∗^p < 0.01).

Immunofluorescence staining for ZEB2 across various stages of organoid development revealed specific nuclear expression appearing earlier in gorilla (Figure 6C). Expression was gradually lost from progenitors as neurogenesis began with a switch at later stages to postmitotic neurons (Figures S6A–S6C). In organoids that were still completing the transition to neurogenic states, we observed a mutually exclusive pattern of expression between ZEB2 and markers of committed RG identity, such as BLBP (Figure 6D) and GLAST (Figure S6D). These data suggest that ZEB2 expression increases in NE cells during their transition to RG cells, but is then lost in fully transitioned RG cells.

To test whether ZEB2 was required for the NE-tNE-RG transition, we generated *ZEB2* mutant human embryonic stem cells (ESCs). CRISPR targeting resulted in recovery of only heterozygous loss-of-function cells (Figures S6E–S6H), suggesting it may be necessary for pluripotency or survival. Heterozygous loss-of-function mutations in *ZEB2* have been described to cause Mowat-Wilson syndrome, a complex disorder that manifests as an array of brain developmental defects with variable penetrance (Mowat et al., 2003). We therefore reasoned that *ZEB2*^+/−^organoids may be informative, both from an evolutionary point of view, and in understanding this human condition. Cytological and RT-PCR analyses confirmed the cells were healthy and expressed key pluripotency marker genes to the same levels as the parental control (Figures S6I and S6J) despite showing a reduction in *ZEB2* mRNA levels (Figure S6J). Western blot analysis of *ZEB2*^+/−^ organoids confirmed a reduction in ZEB2 protein compared to control (Figure S6K) and revealed expected effects on junctional proteins CDH1, CDH2, and OCLN (Figure 6E).

We next tested whether the *ZEB2*^+/−^ mutation led to changes in tissue architecture consistent with a role in NE to RG cell transition. Bright field imaging revealed more prominent and extended neuroepithelial buds (Figure 6F) with overall larger sizes (Figure 6G) and reduced thickness (Figure 6H) consistent with defective NE to RG transition. Furthermore, *ZEB2*^+/−^ organoids showed significantly decreased numbers of TBR2^+^ intermediate progenitor cells at early stages (Figure S6L) that returned to normal at later stages (Figures S6M and S6N), suggesting a potential delay in neurogenesis that would be consistent with a delayed transition to neurogenic RG. We tested the specificity of this phenotype by introducing a doxycycline inducible overexpression construct for *ZEB2-GFP* (Figure S6O) into *ZEB2*^+/−^ cells and selecting clones with proper integration of the expression cassette (Figures S6P and S6Q). *iZEB2* clones of *ZEB2*^+/−^ cells (*ZEB2*^+/−^; *iZEB2*) displayed a rescued phenotype upon doxycycline induction, both in terms of neuroepithelial bud size (Figures 6I and S6R) and TBR2^+^ cell numbers (Figures S6S and S6T).

Staining of early neurogenic organoids showed that although control organoids had redistributed the tight junction epithelial protein OCLN near the apical surface of cells, *ZEB2*^+/−^ mutant organoids retained marked OCLN staining along the entire apico-basal axis of individual progenitor cells (Figure 6J). Together, these findings reveal a specific effect of decreased ZEB2 levels on tight junctions and cell shape of transitioning NE cells that leads to an enlarged neuroepithelium.

### Manipulation of *ZEB2* expression and downstream signaling leads to interspecies phenocopy

**Figure S7:**
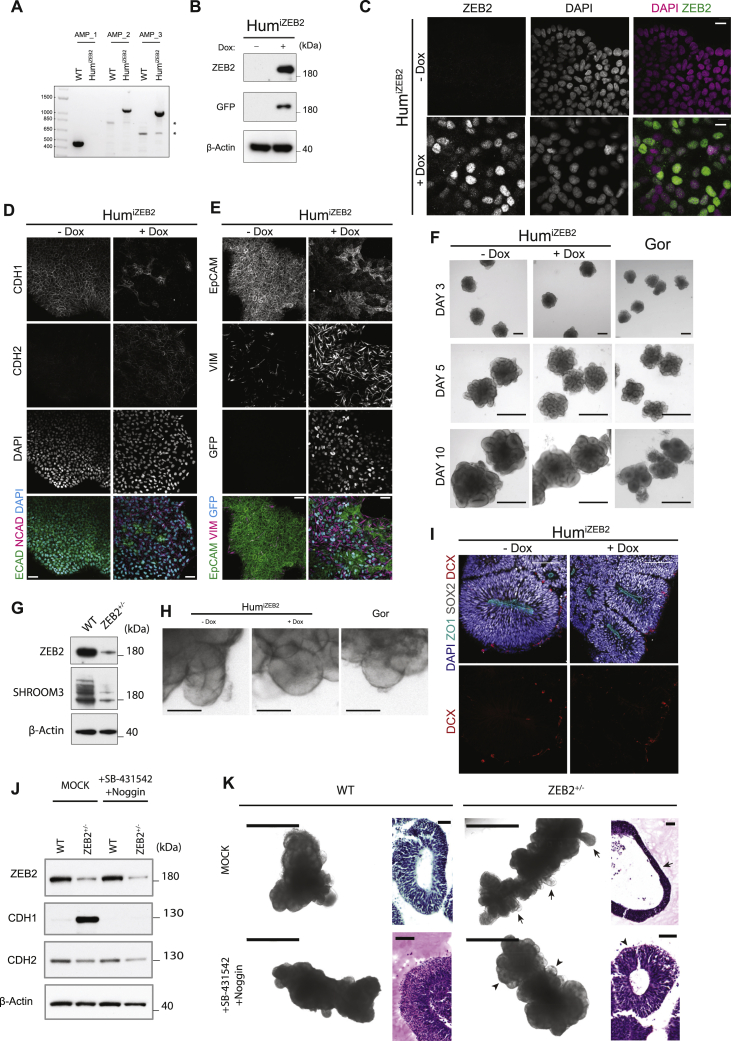
Modulation of ZEB2 and SMAD signaling in human and gorilla cells, related to Figure 7 A. PCR gel showing successful genotyping of the Hum^*iZEB2*^ colony used for all experiments shown based on the PCR genotyping strategy outlined in Figure S6P. The asterisks mark unspecific bands. B. Transgene induction in Hum^*iZEB2*^ cells treated with and without doxycycline for 6 days and assayed by western blot for ZEB2, GFP and β-actin. C. Immunofluorescence images of 6-day induced and uninduced Hum^*iZEB2*^ cells stained for ZEB2 and DAPI, showing that doxycyline induction results in ZEB2 expression and nuclear translocation, without adverse effects on its localization due to tagging with GFP. Scale bar: 20 μm D. Immunofluorescence images of 6-day induced and uninduced Hum^*iZEB2*^ cells stained for DAPI, CDH1 and CDH2. The data reveal a reduction in CDH1 expression and an increase in CDH2 expression following induction. Scale bar: 50 μm. E. Immunofluorescence images of 6-day induced and uninduced Hum^*iZEB2*^ cells stained for GFP, Vimentin and EpCAM. The data reveal a reduction in EpCAM expression and an increase in Vimentin expression following expression of ZEB2-GFP. Scale bar: 50 μm. F. Brightfield images of induced (+ Dox) and uninduced (- Dox) Hum^*iZEB2*^ organoids and gorilla (G1) organoids at days 3, 5 and 10, showing indistinguishable tissue architecture between organoids at day 3, while day 5 and 10 organoids show neuroepithelial buds that are smaller and more rounded in gorilla and *ZEB2* induced (+ Dox) versus uninduced (- Dox) organoids. Scale bar: 200 μm. G. Western blot of day 5 WT and *ZEB2*^*+/−*^ organoids revealing both decreased ZEB2 and SHROOM3 levels in *ZEB2*^*+/−*^ organoids compared to WT control β-Actin was used as loading control. H. Representative bright-field images of uninduced and induced Hum^*iZEB2*^ and gorilla neuroepithelial buds at day 5, used for quantification in Figure 7H. Scale bar: 100 μm. I. Immunofluorescent staining for ZO1, SOX2, DCX and DAPI showing normal tissue morphology and onset of neurogenesis (DCX+ neurons) in uninduced (- Dox) and induced (+ Dox) Hum^*iZEB2*^ organoids at day 10. Scale bar: 100 μm. J. Western blot for ZEB2, CDH1, and CDH2, with β-Actin as loading control, of WT and *ZEB2*^*+/−*^ organoids treated with dual-SMAD inhibitors, or treated with vehicle, for 10 days and assayed at day 12. Note the rescued levels of junctional components CDH1 and CDH2. K. Morphological assessment of WT and *ZEB2*^*+/−*^ organoids treated with dual-SMAD inhibitors, or treated with vehicle, for 10 days and assayed at day 12 by brightfield imaging (left panels) and hematoxylin-eosin staining (right panels). Note the elongated neuroepithelial buds (arrows) in mutant organoids that appear rescued (arrowheads) upon SMAD inhibition. Scale bars: 1 mm (left panels), 50 μm (right panels).

We next tested whether changing *ZEB2* expression dynamics to match that of the gorilla would be sufficient to trigger an earlier transition to tNE cells in human organoids. We generated an otherwise wild-type human cell line harboring the inducible *ZEB2-GFP* described above (Figure S7A). We observed robust and tight expression upon induction, both by western blot and immunofluorescent staining (Figures S7B and S7C). Following doxycycline treatment in stem cells, we observed a reduction in EpCAM and CDH1 staining accompanied by an increase in VIM and CDH2 staining (Figures S7D and S7E) relative to the uninduced control.

**Figure 7:**
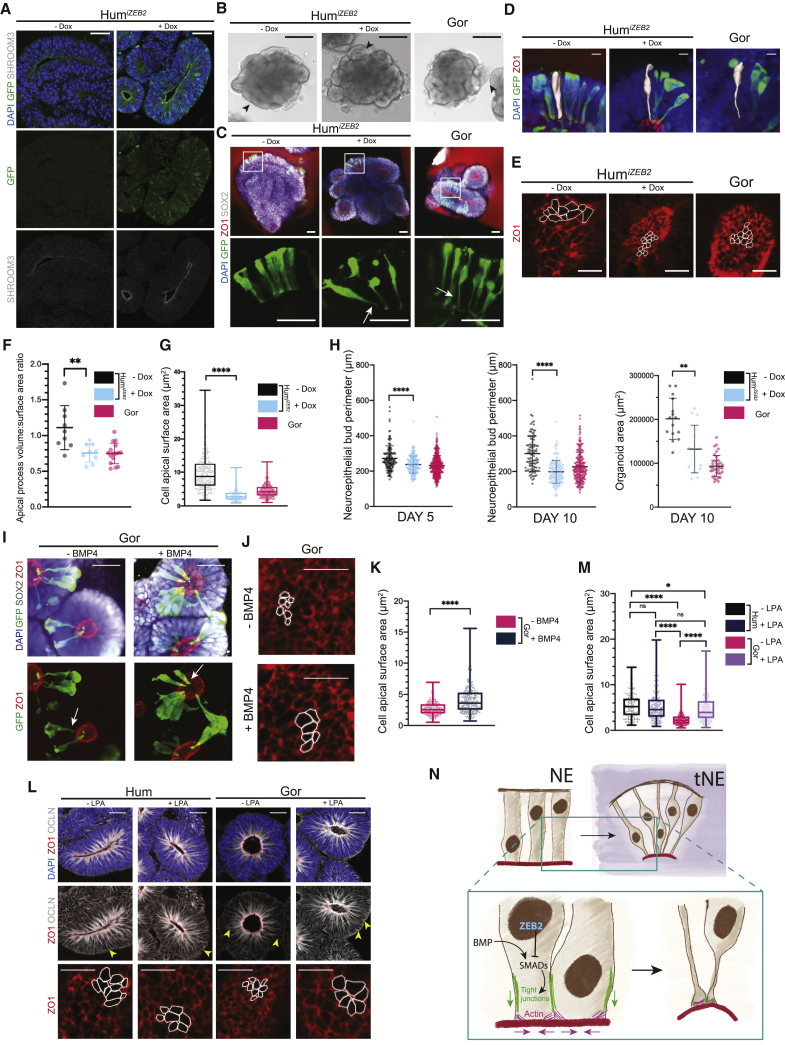
ZEB2-driven junctional remodeling and apical constriction dictate species-specific timing of NE transition (A) Immunofluorescent staining of uninduced (− Dox) and induced (+ Dox) Hum^*iZEB2*^ organoids for GFP and SHROOM3. Note the expression of ZEB2-GFP and apical accumulation of SHROOM3 in induced organoids. Scale bar, 50 μm. (B) Representative bright field images of day 5 Hum^*iZEB2*^ and gorilla organoids. Induced (+ Dox) Hum^*iZEB2*^ organoids show smaller neuroepithelial buds (arrowheads) that are more round in shape, similar to gorilla (G1), while uninduced (− Dox) show more elongated structures typical of human. Scale bar, 200 μm. (C) Immunofluorescence images through day 5 whole mount Hum^*iZEB2*^ uninduced (− Dox), induced (+ Dox) and gorilla (G1) organoids stained for GFP, ZO1, and SOX2. Sparse labeling with viral GFP shows *ZEB2* induction triggers the constriction of apical processes (arrows) in progenitor cells, similar to gorilla at day 5. Scale bar, 50 μm. (D) Representative immunofluorescence images through whole mount day 5 uninduced (− Dox), induced (+ Dox) Hum^*iZEB2*^ and gorilla (G1) organoids with superimposed individual segmented GFP+ progenitor cells (white) showing their 3D morphology. Note the thinning of apical processes observed upon *ZEB2* induction. Scale bar, 10 μm. (E) Immunofluorescent staining for ZO1 on the surface of apical lumens showing the apical surface areas of individual progenitor cells in day 5 Hum^*iZEB2*^ uninduced (− Dox), induced (+ Dox) and gorilla (G1) organoids. Perimeters of some individual progenitor cells of day 5 organoids are delineated in white. Scale bar, 10 μm. (F) Quantification of the volume as normalized to surface area of the apical processes of induced (+ Dox) versus uninduced (− Dox) Hum^*iZEB2*^ neural progenitor cells on day 5. The apical processes of segmented cells directly below the cell body were used for quantification. Gorilla day 5 measurements from Figure 2E are included for comparison. Mean apical process volume:surface area ratio: Hum^*iZEB2*^ − Dox = 1.11; Hum^*iZEB2*^ + Dox = 0.76. ^∗∗^p < 0.01, Mann-Whitney U, two-tailed, n (− Dox and + Dox) = 9 cells. Error bars are SD. (G) Quantification of the surface area of individual delineated ZO1 cell perimeters as shown in (E). Gorilla measurements from two cell lines (G1, G2) combined are shown for comparison. Mean apical surface area/cell: Hum^*iZEB2*^ − Dox = 9.62 μm^2^, Hum^*iZEB2*^ + Dox = 3.08 μm^2^, and gorilla (G1,G2) = 4.50 μm^2^. ^∗∗∗∗^p < 0.0001, Mann-Whitney U, two-tailed, n (− Dox) = 180 cells from 8 organoids, n (+ Dox) = 199 cells from 8 organoids, both from 2 independent batches, box and whisker plots show median with min-max values, data points represent individual cells. (H) Quantification of perimeters of neuroepithelial buds from bright field images at days 5 and 10, and overall organoid size at day 10. Gorilla (G1,G2) measurements were combined and included for comparison. Day 5 mean neuroepithelial bud perimeter: Hum^*iZEB2*^ − Dox = 272 μm, Hum^*iZEB2*^ + Dox = 237 μm, and gorilla (G1,G2) = 232 μm. ^∗∗∗∗^p < 0.0001, Mann-Whitney U, two-tailed, n (− Dox) = 142 neuroepithelial buds from 41 organoids from 3 independent batches; n (+Dox) = 195 neuroepithelial buds from 38 organoids from 3 independent batches; n (G1,G2) = 555 neuroepithelial buds from 114 organoids from 16 independent batches. Day 10 mean neuroepithelial bud perimeter: Hum^*iZEB2*^ − Dox = 300 μm, Hum^*iZEB2*^ + Dox = 198 μm, and gorilla (G1,G2) = 227 μm. Day 10 mean organoid area: Hum^*iZEB2*^ − Dox = 201,434 μm^2^, Hum^*iZEB2*^ + Dox = 132,325 μm^2^, and gorilla (G1,G2) = 93,447 μm^2^. ^∗∗∗∗^p < 0.0001, Mann-Whitney U, two-tailed, n (− Dox day 10) = 15 organoids and 106 neuroepithelial buds from 3 independent batches, n (+ Dox day 10) = 15 organoids and 149 neuroepithelial buds from 3 independent batches, error bars are SD. (I) Representative immunofluorescence images showing the effect of BMP4 on the morphology of neural progenitor cells revealed by sparse viral labeling with GFP on day 5 untreated (− BMP4) and treated (+ BMP4) gorilla (G1) organoids with staining for GFP, SOX2, and DAPI. Arrows indicate the apical process. Scale bar, 40 μm. (J) Immunofluorescent staining for ZO1 showing apical surface areas of individual progenitor cells from BMP4-treated (+ BMP4) and untreated (− BMP4) gorilla (G1) organoids at day 5. Perimeters of some individual progenitor cells are delineated in white. Scale bar, 10 μm. (K) Quantification of individual delineated ZO1 cell perimeters as shown in (J). Mean apical surface area/cell: gorilla − BMP4 = 2.72 μm^2^ and gorilla + BMP4 = 4.13 μm^2^. ^∗∗∗∗^p < 0.0001, Mann-Whitney U, two-tailed, n (− BMP4) = 301 cells from 8 organoids from 2 independent batches, n (+ BMP4) = 326 cells from 8 organoids from 2 independent batches, box and whisker plots are median with min-max values, data points represent individual cells. (L) Immunofluorescence images of human (H9) and gorilla (G1) day 5 organoids untreated (− LPA) and treated (+ LPA) with staining for OCLN, ZO1, and DAPI. LPA treatment in gorilla results in increased OCLN distribution along the apicobasal axis of cells (arrowheads) and expanded apical surfaces of cells (ZO1, bottom panel). Scale bar, 40 μm (upper panels), 10 μm (bottom panels). (M) Quantification of individual delineated ZO1 cell perimeters as shown in (L). Mean apical surface area/cell: human − LPA = 5.36 μm^2^, human + LPA = 5.25 μm^2^, gorilla − LPA = 2.44 μm^2^, and gorilla + LPA = 4.73 μm^2^. ^∗^p < 0.05 ^∗∗∗∗^p < 0.0001, Kruskal-Wallis and post hoc Dunn’s multiple comparisons test, n (human − LPA) = 146 cells from 3 organoids of 1 batch, n (human + LPA) = 200 cells from 3 organoids of 1 batch, n (gorilla − LPA) = 375 cells from 7 organoids and 2 independent batches, n (gorilla + LPA) = 457 cells from 10 organoids and 2 independent batches, box and whisker plots show median with min-max values, data points represent individual cells. (N) Schematic summarizing the morphological changes that occur in neural progenitor cells as they transition from NE to tNE cells (purple background). ZEB2 is highlighted as a driver, which acts through BMP-responsive SMADs to downregulate epithelial features, notably tight-junction proteins (TJs, green), and involves apical constriction through rearrangements in the actin cytoskeleton (actin, magenta). See also Figure S7.

Organoids from these inducible *ZEB2* human (Hum^*iZEB2*^) cells were treated with doxycycline to induce *ZEB2* expression at an earlier stage (Figure 7A). Examination of tissue architecture at day 3 revealed indistinguishable morphology between uninduced Hum^*iZEB2*^, induced Hum^*iZEB2*^, and gorilla organoids (Figure S7F). By day 5, however, neuroepithelial buds generated in induced Hum^*iZEB2*^ organoids were more similar to gorilla in shape, appearing smaller and more rounded, whereas uninduced Hum^*iZEB2*^ organoids exhibited buds with more elongated shapes typical of human organoids (Figures 7B and S7F). This change in morphology was also accompanied by earlier expression and localization of SHROOM3 (Figures 7A and S7G), the actin regulator identified to exhibit precocious expression in gorilla (Figure 5D).

Sparse viral labeling with GFP revealed that NE cells of induced Hum^*iZEB2*^ organoids were less columnar and more tNE-like in shape than uninduced cells, with a thinning of apical and basal processes that was highly similar to gorilla tNE cells at the same time point (Figure 7C). Changes in cell shape and apical constriction were quantified by cell segmentation (Figure 7D) and ZO1 staining of the apical surface (Figure 7E), revealing a remarkably similar apical constriction in human *ZEB2* overexpressing organoids to gorilla organoids (Figures 7F and 7G). We further tested the effect on tissue size by measuring neuroepithelial bud perimeter and organoid area (Figures 7H and S7H), revealing a phenotype reminiscent of the nonhuman ape. Staining of later stages revealed normal neurogenesis (Figure S7I), demonstrating that these effects seemed to specifically affect the timing and not the identity or fate of progenitors. These findings suggest that premature expression of *ZEB2* in human organoids is sufficient to recapitulate the precocious tNE cell shape change seen in gorilla organoids.

ZEB2, also known as SMAD interacting protein 1 (SIP1), is a known regulator of SMAD signaling that leads to downstream effects on cell-cell junction proteins including OCLN and CDH1 and 2 (Vandewalle et al., 2005). We therefore tested whether SMAD signaling may also be responsible for the effects of ZEB2 in this context by performing rescue experiments with SMAD inhibitors in the context of *ZEB2*^+/−^ mutation. We observed a rescue of the effects of ZEB2 on expression of junction protein CDH1 (Figure S7J) as well as rescued tissue architecture (Figure S7K), suggesting SMAD signaling may be responsible for the EMT effects of ZEB2 during the NE to RG transition.

We next took advantage of this information to see whether gorilla organoids could be encouraged to take on a more human-like phenotype by manipulating SMAD signaling. We focused on activation of BMP-responsive SMADs, because this pathway has previously been shown to inhibit EMT (Amarnath and Agarwala, 2017), and ZEB2 can inhibit BMP-responsive SMADs in other neuronal contexts (Menuchin-Lasowski et al., 2020). Application of BMP4 on gorilla organoids when they would normally begin transitioning to tNE cell morphologies led to the appearance of cells with more dilated apical processes (Figure 7I) and enlarged apical contacts (Figures 7J and 7K), reminiscent of human NE cells. Thus, by activating downstream SMADs, we were able to override the EMT processes that would normally promote acquisition of tNE morphology at this stage.

Finally, because we observed a species-specific change in actin-associated apical constriction and SHROOM3 expression that was also responsive to ZEB2 (Figures 7A and S7G), we used an orthogonal approach to manipulate apical constriction through the use of lysophosphatidic acid (LPA), a molecule previously shown to enlarge apical contacts of NE cells *in vitro* (Medelnik et al., 2018). Application of LPA on gorilla organoids at the onset of NE transition led to morphologies more closely mimicking the human phenotype (Figure 7L) with enlarged apical contacts (Figure 7M) and more extended OCLN staining along the apicobasal axis (Figure 7L). Together, these findings point to a close interaction between cell-cell junctions and apical constriction, both of which are regulated by ZEB2 to drive the change in cell shape that characterizes this newly described transition state (Figure 7N).

## Discussion

These results provide insight into how NE cells transition into neurogenic RG cells, highlighting an intermediate tNE cell stage, hitherto unidentified in the mammalian neocortex. Interestingly, a transitioning NE cell type has been described in *Drosophila* optic primordia with features analogous to those we have observed in human and ape organoids (Orihara-Ono et al., 2011), raising the intriguing possibility that such a transition may be evolutionarily conserved. Furthermore, the temporal progression of events and RNA-seq reveal a shift in cell morphology preceding other changes associated with RG identity, namely expression of glial markers and production of neurons. Previous findings have similarly revealed an important role for cell morphology during neurogenesis, but in the context of proliferating basal progenitors (Kalebic et al., 2019). Thus, cell shape may be a key initiator in general during these developmental transitions.

The protracted shift to the tNE morphotype in humans, coupled with a shorter cell cycle, is particularly intriguing with regard to human brain evolution, as it may explain the linear scaling of the human brain compared with apes (Herculano-Houzel, 2009). An evolutionary modification at such an early developmental stage would be expected to affect all subsequent steps of neurogenesis and lead to a proportional increase in all neuron types. Indeed, mathematical modeling reveals an expected increase in neurons, and staining for neurons even at early stages reveals increased numbers in human organoids (Figure S2A). Furthermore, cytoarchitecturally, this would be expected to lead not to a thickened cortical gray matter, but rather to a more expanded brain in the tangential dimension, which is precisely what is seen in comparative neuroanatomy to other apes (Donahue et al., 2018; Hutsler et al., 2005).

Together, these findings reveal a mechanism for evolutionary human brain expansion, and highlight ZEB2 as a key regulator. ZEB2 was previously shown to be involved in early germ layer specification (Chng et al., 2010; Eisaki et al., 2000; Stryjewska et al., 2017), neural tube morphogenesis (Miyoshi et al., 2006), and neural crest differentiation (Yasumi et al., 2016). In the cortex specifically, ZEB2 has been shown to regulate the timing of cortical laminar fate determination (Epifanova et al., 2019; Parthasarathy et al., 2014; Seuntjens et al., 2009) as well as neocortical axon outgrowth (Srivatsa et al., 2015). *ZEB2*’s previous identification as the causative gene in Mowat-Wilson syndrome (Mowat et al., 2003) further highlights its importance in human neurodevelopment. Interestingly, patients with Mowat-Wilson syndrome also exhibit ventriculomegaly (Garavelli et al., 2017), or enlarged ventricles, a phenotype paralleled by the enlarged neuroepithelial buds seen in *ZEB2*^+/−^ organoids.

ZEB2 is also well-known as a key regulator of EMT (Fardi et al., 2019). In many respects, the NE-tNE-RG transition resembles a partial EMT process with a progressive change in cell morphology, apical constriction, and a reduction in cell-cell junctions (Pastushenko and Blanpain, 2019). Furthermore, apical constriction is the first morphological change associated with EMT in several tissues (Gracia et al., 2019; Saunders and McClay, 2014; Williams et al., 2012), and changes in epithelial cell-cell junctions are linked to cytoskeletal changes (Morris and Machesky, 2015) with several reports having shown cross-regulation between ZEB2 and the actin cytoskeleton (Wiles et al., 2013; Yuan et al., 2019). Our findings add to this and support a link between ZEB2 and cytoskeletal targets with SHROOM3 as a key player (Chu et al., 2013).

Overall, our findings suggest that a relatively simple evolutionary change in cell shape, regulated by the fundamental process of EMT, can have major consequences in brain evolution.

### Limitations of study

This study was performed using neural organoids, and like all models, such organoids will never be a perfect representation of the actual brain *in vivo*. However, because it is impossible to perform experiments on living human or ape embryos, organoids represent an important tool for both comparative studies and functional interrogation. Nonetheless, further examination using *in vivo* approaches, such as transgenic mice, or even non-invasive imaging of ape embryos would be highly informative in the future.

## STAR★Methods

### Key resources table

**Table:** 

REAGENT or RESOURCE	SOURCE	IDENTIFIER
**Antibodies**		

mouse anti-β-actin	Abcam	Cat# ab8226, RRID:AB_306371
mouse anti-ZEB2	Origene	Cat# TA802113, RRID:AB_2616296
mouse anti-CDH2	BD Biosciences	Cat# 610920, RRID:AB_2077527
mouse anti-CDH1	BD Biosciences	Cat# 610181, RRID:AB_397580
rabbit anti-SOX2	Abcam	Cat# ab97959, RRID:AB_2341193
sheep anti-TBR2	R&D Systems	Cat# AF6166, RRID:AB_10569705
chicken anti-GFP	Thermo Fisher Scientific	Cat# A10262, RRID:AB_2534023
rabbit anti-OCLN	Abcam	Cat# ab31721, RRID:AB_881773
rabbit anti-GLAST	Abcam	Cat# ab416, RRID:AB_304334
rat anti-CTIP2	Abcam	Cat# ab18465, RRID:AB_2064130
mouse anti-HuC/D	Life Technologies	Cat# A-21271, RRID:AB_221448
rabbit anti-EMX1	Atlas Antibodies	Cat# HPA006421, RRID:AB_1078739
rabbit anti-EMX1	Origene	TA325087
rabbit anti-BLBP	Abcam	Cat# ab32423, RRID:AB_880078
goat anti-DCX	Santa Cruz Biotechnology	Cat# sc-8067, RRID:AB_2088491
mouse anti-ZO1	BD Biosciences	Cat# 610966, RRID:AB_398279
rabbit anti-GFP	Abcam	Cat# ab290, RRID:AB_303395
rabbit anti-EpCAM	Abcam	Cat# ab71916, RRID:AB_1603782
mouse anti-Vimentin (V9)	Santa Cruz Biotechnology	Cat# sc-6260, RRID:AB_628437
rabbit anti-PAX6	Abcam	Cat# ab195045, RRID:AB_2750924
rabbit anti-SHROOM3	Atlas Antibodies	Cat# HPA047784, RRID:AB_2680155
mouse anti-GAPDH	Abcam	Cat# ab8245, RRID: AB_2107448
mouse anti-TUJ1	Biolegend	Cat# 801213, RRID: AB_2728521
HRP-linked goat anti-rabbit	Dako	Cat# P0448, RRID:AB_2617138
HRP-linked rabbit anti-mouse	Dako	Cat# P0161, RRID:AB_2687969
Alexa Fluor 594 Phalloidin	Thermo Fisher Scientific	Cat# A12381, RRID:AB_2315633
AlexaFluor 488, donkey anti-chicken IgY (IgG) (H+L)	Jackson ImmunoResearch Labs	Cat# 703-545-155, RRID:AB_2340375
AlexaFluor 488, 568, 647 donkey anti-rabbit IgG (H+L)	Life Technologies	Cat#A32790, RRID: AB_2762833; A10042, RRID: AB_2534017; A31573, RRID: AB_2536183
AlexaFluor 488, 568, 647 donkey anti-mouse IgG (H+L)	Life Technologies	Cat#A21202, RRID: AB_141607; A10037, RRID: AB_2534013; A31571, RRID: AB_162542
AlexaFluor 568, 647 donkey anti-sheep IgG (H+L)	Life Technologies	Cat#A21099, RRID: AB_2535753; A21448, RRID: AB_2535865
AlexaFluor 488, 568, 647 donkey anti-goat IgG (H+L)	Life Technologies	Cat#A11055, RRID: AB_2534102; A11057, RRID: AB_2534104; A21447, RRID: AB_2535864

**Bacterial and virus strains**		

CytoTune emGFP Sendai fluorescence reporter	Thermo Fisher Scientific	Cat#A16519

**Chemicals, peptides, and recombinant proteins**		

Matrigel	Corning	Cat#354234
StemFlex culture media	Thermo Fisher Scientific	Cat#A3349401
Lymphoprep	Stem Cell Technologies	Cat#07801
Ascorbic acid	Sigma	Cat#A4403
SCF	Miltenyi	Cat#130-096-692
IL-3	Invitrogen	Cat#PHC0035
EPO	R&D systems	Cat#287-TC-500
IGF-1	Miltenyi	Cat#130-093-885
Dexamethasone	Sigma	Cat# D8893
iPS Boost Supplements II	Millipore	Cat#SCM094
FBS	Thermo Fisher Scientific	Cat#10270106
TrypLE	Thermo Fisher Scientific	Cat#12563029
Rock Inhibitor Y27632	Merck	Cat#688000-5
Accutase	Sigma	Cat#A6964
DMEM F-12	Thermo Fisher Scientific	Cat#11330032
Neurobasal Medium	Invitrogen	Cat#21103049
MEM-NEEA	Sigma	Cat#M7145
GlutaMAX	Thermo Fisher Scientific	Cat#35050038
Penicillin-Streptomycin	Sigma	Cat#P0781
N2	Thermo Fisher Scientific	Cat#17502048
B27	Thermo Fisher Scientific	Cat#17504044
B27 minus vitamin A	Thermo Fisher Scientific	Cat#12587010
β-Mercaptoethanol	Life Technologies	Cat#31350-010
Knockout Serum Replacement	Thermo Fisher Scientific	Cat#10828028
bFGF (FGF2)	Peprotech	Cat#100-18B
Insulin	Sigma	Cat#I9278
Heparin	Sigma	Cat#H3149
CHIR99021	Tocris	Cat#4423
LPA	Tocris	Cat#325465-93-8
BMP4	R&D Systems	Cat#314-BP
Noggin	R&D Systems	Cat#6057-NG
SB-431542	R&D Systems	Cat#1614
Halt Protease inhibitor cocktail	Thermo Fisher Scientific	Cat#78430
PhosSTOP	Sigma	Cat#4906845001
DAPI	Life Technologies	Cat#D1306
Prolong Diamond Antifade mountant	Invitrogen	Cat#P36965
2-methylbutane	Sigma	Cat#M32631
TRIZOL reagent	Thermo Fisher Scientific	Cat#15596026
DAPI	Life Technologies	Cat#D1306

**Critical commercial assays**		

Cerebral Organoid kit	Stem Cell Technologies	Cat#08570, 08571
Direct-zol-96 RNA kit	Zymo Research	Cat#R2055
NEBNext Ultra II Directional RNA Library Prep Kit for Illumina	New England Biolabs	Cat#E7760
NEBNext Multiplex Oligos for Illumina	New England Biolabs	Cat#E7600
Qubit dsDNA HS Assay Kit	Thermo Fisher Scientific	Cat#Q32851
Quick Start Bradford Dye Reagent	Bio-Rad	Cat#5000205
Amersham Hybond PVDF blotting membrane	GE Healthcare	Cat#10600023
ECL Prime enhanced chemoluminescent detection reagent	GE Healthcare	Cat#RPN2232
SuperSignal West Femto chemoluminescent substrate	Thermo Fisher Scientific	Cat#34094
Human Pluripotent Stem Cell Assessment Primer Pair Panel	R&D Systems	Cat#SC012
GoTaq Green Master Mix	Promega	Cat#9PIM712
Q5 High Fidelity 2X Master Mix	New England Biolabs	Cat#M0492S
ddPCR Supermix for Probes	Bio-Rad	Cat#1863024
SuperScript III first-strand synthesis supermix	Thermo Fisher Scientific	Cat#18080400
Droplet Generation Oil for Probes	Bio-Rad	Cat# 1863005
Human Stem Cell Nucleofector Kit 1 (Lonza, VPH-5012	Lonza	Cat#VPH-5012
RevitaCell	Thermo Fisher Scientific	Cat#A2644501

**Deposited data**		

Raw and analyzed RNA-sequencing data	This paper	NCBI GEO: GSE153076
Human reference genome NCBI build 38, GRCh38	Genome Reference Consortium	https://www.ncbi.nlm.nih.gov/projects/genome/assembly/grc/human/
Gorilla reference genome, Kamilah_GGO_v0	University of Washington	https://www.ncbi.nlm.nih.gov/assembly/GCF_008122165.1/

**Experimental models: Cell lines**		

Human: H9 ES	WiCell	WA09
Human: IMR-90 iPS	WiCell	iPS(IMR90)-4
Chimpanzee: Chmp iPS	Gallego Romero et al., 2015	C3651
Gorilla: G1 iPS	Wunderlich et al., 2014	goiPSC clone 1
Gorilla: G2 iPS	This paper	N/A
Human: H9 *ZEB2*^*+/−*^ ES	This paper	N/A
Human: H9 *ZEB2*^*+/−*^*; iZEB2* ES	This paper	N/A
Human: Hum^*iZEB2*^ (H9^*iZEB2*^*)* ES	This paper	N/A
Mouse: MEF feeders	GIBCO	Cat#A34181

**Oligonucleotides**		

See Table S1		

**Recombinant DNA**		

AAVS1-Neo-TRE-CMV-Cre-rtTA	This paper	RRID: Addgene_165457
AAVS1-Puro-CAG-fl-STOP-fl-ZEB2-GFP-Flag-WPRE-poly(A)	This paper	RRID: Addgene_165456
pT2-CAG-fGFP	Addgene	RRID: Addgene_108714
Puro-Cas9 donor	Addgene	RRID: Addgene_58409
pAAV-Neo_CAG-Cas9	Addgene	RRID: Addgene_86698
AAVS1-Neo-M2rtTA	Addgene	RRID: Addgene_60843
pCAG-Cre	Addgene	RRID: Addgene_13775
pSpCas9(BB)-2A-GFP (PX458)	Addgene	RRID: Addgene_48138
pENTRY221-ZEB2 ORF	Thermo Fisher Scientific	Ultimate ORF clone number: IOH53645
pSpCas9n (BB) (PX460)	Addgene	RRID: Addgene_48873

**Software and algorithms**		

Fiji	Schindelin et al., 2012	https://imagej.net/Fiji
Prism v8.4.2	GraphPad	https://www.graphpad.com/
R v3.5.2	R Core Team, 2018	http://www.R-project.org/
MATLAB R2019a	Mathworks	https://www.mathworks.com/products/matlab.html
Imaris v9.2	Oxford Instruments	https://imaris.oxinst.com/
MaMuT v0.27.0	Wolff et al., 2018	https://imagej.net/MaMuT
PRAGUI	MRC LMB	https://github.com/lmb-seq/PRAGUI
Trim Galore! v0.6.3	Krueger, 2012	https://github.com/FelixKrueger/TrimGalore/releases
Cutadapt v2.4	Martin, 2011	https://cutadapt.readthedocs.io/en/stable/
FASTQC v0.11.5	Andrews, 2010	https://github.com/s-andrews/FastQC
HISAT2 v2.0.0-beta	Kim et al., 2015	http://daehwankimlab.github.io/hisat2/
HTSeq v0.11.2	Anders et al., 2015	https://htseq.readthedocs.io/en/master/
g:Profiler	Reimand et al., 2007	https://biit.cs.ut.ee/gprofiler/gost
TCseq	Wu and Gu, 2020	https://rdrr.io/bioc/TCseq/f/inst/doc/TCseq.pdf
TBR2+ cell counter	This paper	https://github.com/StefanoLG92/FIJI-macros
Organoid lumen segmentation	This paper	https://github.com/esriis/organoid-lumen-segmentation
QuantaSoft Software	Bio-Rad	https://www.bio-rad.com/

### Resource availability

#### Lead contact

Further information and requests for resources and reagents should be directed to and will be fulfilled by the Lead Contact, Madeline Lancaster (madeline.lancaster@mrc-lmb.cam.ac.uk).

#### Materials availability

All cell lines, except Gorilla gorC1 iPS cells, were previously published or available from commercial sources. gorC1 cells are available from the Lead Contact subject to institutional MTA and CITES regulations. Newly generated plasmid constructs AAVS1-Neo-TRE-CMV-Cre-rtTA and AAVS1-Puro-CAG-fl-STOP-fl-ZEB2-GFP-Flag-WPRE-poly(A) are available through Addgene (165457 and 165456, respectively).

#### Data and code availability

The accession number for the RNaseq data reported in this paper is NCBI GEO: GSE153076. The custom code used for image analysis and lumen segmentation is publicly available at https://github.com/esriis/organoid-lumen-segmentation. The custom code used for segmentation and counting of TBR2+ cells is publicly available at (https://github.com/StefanoLG92/FIJI-macros).

### Experimental model and subject details

#### Cell lines

One human ESC line (H9), one human iPSC line (iPS(IMR90)-4, shortened to IMR-90 in this study), one chimpanzee iPSC line (C3651, shortened to Chmp) and two gorilla iPSC lines (goiPSC clone 1 and gorC1, shortened to G1 and G2 respectively) were used in this study. All cell lines were female. H9 (WA09) and iPS(IMR90)-4 were purchased from WiCell. C3651 was a gift from Yoav Gilad (Gallego Romero et al., 2015). goiPSC clone 1 was previously published (Wunderlich et al., 2014). We generated gorC1 using peripheral blood mononuclear cells (PBMCs) isolated from leftover blood collected from a female western lowland gorilla during a routine check-up at Twycross zoo. Briefly, PBMCs were isolated using Lymphoprep (Stem Cell Technologies, 07801), followed by expansion for 9 days in Expansion Media composed of Stemspan H3000 (Stem Cell Technologies, 09850) supplemented with 50 μg/ml ascorbic acid (Sigma, A4403), 50 ng/ml SCF (Miltenyi, 130-096-692), 10 ng/ml IL-3 (Invitrogen, PHC0035), 2 U/ml EPO (R&D Systems, 287-TC-500), 40 ng/ml IGF-1 (Miltenyi, 130-093-885), 1 μM Dexamethasone (Sigma, D8893-1MG). On day 10, expanded PBMCs were transduced with non-integrating Sendai reprogramming virus (Cytotune 2.0, Thermo Fisher, A16517) according to manufacturer’s instructions added to the Expansion Media. The next day, the media was changed and two days later the cells were collected and plated on gamma irradiated MEF feeders (GIBCO, A34181) in human ES media (DMEM/F12 (Invitrogen, 11330-032) with 20% knockout serum replacement (Invitrogen, 10828-028), 1:100 Glutamax (Invitrogen 35050-038), 1:100 NEAA (Sigma, M7145), 3.5 μl/500ml 2-mercaptoethanol, 20 ng/ml bFGF (Peprotech, 100-18B)) supplemented with ascorbic acid, SCF, IL-3, EPO, IGF-1, and Dexamethasone at the above concentrations along with iPS Boost Supplements II (Millipore, SCM094). Two days later (5 days after induction), media was changed to human ES media without PBMC supplements but with Boost Supplements. Cells were maintained in hES + Boost until colonies began to form (10 days after induction). The small colonies were then split off MEFs 15 days after induction using collagenase and a cell lifter and replated on Matrigel (Corning) coated dishes in StemFlex (Thermo Fisher, A334901) for further selection and propagation. Human ESCs used in this project were approved for use in this project by the UK Stem Cell Bank Steering Committee and iPSCs and ESCs were approved by an ERC ethics committee and are registered on the Human Pluripotent Stem Cell Registry (hpscreg.eu). Nonhuman primate cells were approved by the Animal Welfare and Ethical Review Body (AWERB) of the MRC-LMB. RT-PCR (refer to the *‘*PCR analysis’ section of the methods) confirmed pluripotent gene expression (Figure S1F). All cell lines were maintained in StemFlex on Matrigel coated plates. Cells were passaged every 3-4 days using 0.7 mM EDTA. HEK293 cells were a gift from Dr Harvey McMahon and were cultured in high-glucose DMEM supplemented with GlutaMAX (Thermo Fisher, 31966047) and 10% FBS (Thermo Fisher, 10270106) and passaged once a week using TrypLE (Thermo Fisher, 12563029).

Commercial cell lines (H9 and iPS(IMR90)-4) were authenticated by the provider. Nonhuman primate cells were not authenticated, but were established by the authors, apart from C3651.

### Method details

#### Plasmid Constructs

All oligos used for cloning are listed in Table S1. All PCR amplification steps were done using Q5 polymerase (NEB, M049L) and TOP10 chemically competent *E. coli* (Thermo Fisher, C404010) were used for transformation. In order to generate the construct pT2-CAG-fl-STOP-fl-fGFP, a fl-STOP-fl cassette was ligated into the pT2-CAG-fGFP (Addgene plasmid #108714) at EcoRI and Ascl restriction sites. The construct AAVS1-Puro-CAG-fl-STOP-fl-Cas9 was generated as follows; the backbone was amplified from the Puro-Cas9 donor plasmid (a gift from Dr Danwei Huangfu, Addgene plasmid #58409) (González et al., 2014) with primers AAVS1_CAG_fl_STOP_fl_F & R to introduce MluI (NEB, R3198S) and KpnI (NEB, R3142S) restriction sites and blunt-end ligated to the CAG-fl-STOP-fl cassette, excised from the construct pT2-CAG-fl-STOP-fl-fGFP with restriction enzymes AflI (NEB, R0520S) and AscI (NEB, R0558S) and blunted with the Quick Blunting Kit (NEB, E1201). The resulting intermediate plasmid was then digested with MluI and KpnI and ligated to the cassette containing Cas9 and the bGH-poly(A) sequence amplified from pAAV-Neo_CAG-Cas9 (a gift from Dr Ludovic Vallier, Addgene plasmid #86698) (Bertero et al., 2016) with primers Cas9_β_globin_pA_F & R. All ligations to generate this construct were done with the Quick Ligation Kit (NEB, M2200S) and the final construct was sequence validated. The construct AAVS1-Neo-TRE-CMV-CRE-rtTA was generated as follows; the tight TRE promoter was amplified from the Puro-Cas9 donor plasmid (Addgene plasmid #58409) with primers TRE_F &R and inserted into the EcoRV restriction site of AAVS1-Neo-M2rtTA (a gift from Dr Rudolf Jaenisch, Addgene plasmid #60843) (DeKelver et al., 2010) to produce the intermediate construct AAVS1-Neo-M2rtTA-TRE. A fragment containing CMV-2TO-MCS-bGH-poly(A) was PCR amplified from pcDNA4/TO (Thermo Fisher, V102020) using primers CMV_bGH_F and _R, and inserted into the EcoRV restriction site of AAVS1-Neo-M2rtTA. The fragment comprised between the SalI and AleI restriction sites was replaced with the Cre recombinase cDNA amplified from pCAG-Cre (a gift from Dr Connie Cepko, Addgene plasmid #13775) (Matsuda and Cepko, 2007) using primers Cre_SalI_F and Cre_KpnI_R. From the resulting plasmid a fragment containing CRE-bGH-poly(A) was excised with restriction enzymes SalI (NEB, R3138S) and AleI (NEB, R0634) and inserted into AAVS1-Neo-M2rtTA-TRE digested with the same restriction enzymes. All ligations to generate this construct were done with the Quick Ligation Kit (NEB, M2200S). The final construct was sequence validated. The PX458-AAVS1 construct used to target the AAVS1 locus was generated by cloning the AAVS1_sgRNA sequence into pSpCas9(BB)-2A-GFP (PX458) (a gift from Dr Fang Zhang, Addgene plasmid #48138) as previously described by Ran et al. (2013). In order to generate the construct for inducible *ZEB2* expression from the AAVS1 site the coding sequence of the human *ZEB2* transcript variant 1 (NM_014795.3) was purchased as an ultimate ORF clone in the pENTRY221 (Thermo Scientific, #IOH53645). The *ZEB2* ORF was amplified with primers ZEB2_IndOE_F & _R and tagged with GFP-Flag by Gibson assembly into the pT2-CAG-MCS-2A-Puro plasmid linearized by restriction digestion with EcoRI (NEB, R3101S) and AgeI (NEB, R3552S). The GFP fragment was amplified and C-terminally Flag-tagged using primers GFP_IndOE_F & R and the construct pT2-CAG-fGFP (Addgene plasmid #108714) as template. While the original design aimed to fuse ZEB2 to GFP via a 4x(GGGGS)-linker motif, Gibson assembly resulted in motif duplication and produced a 6x(GGGGS)-linker between the two coding sequences. The resulting plasmid, pT2-CAG-ZEB2-GFP-Flag-2A-Puro, was used as template in a PCR reaction with primers ZEB2_GFP_Flag_IndOE_F & _R. The resulting fragment encoding ZEB2-GFP-Flag was then digested with restriction enzymes AgeI (NEB, R3552S) and KpnI (NEB, R3142S) and ligated into the CAGs-MCS-Enhanced Episomal Vector (EEV) (System Biosciences, EEV600A-1) linearized with AgeI and KpnI, using T4 ligase. Next, using primers ZEB2_AAVS1 IndOE_F &_R a fragment containing ZEB2-GFP-Flag-WPRE-poly(A) and overhangs containing MluI and FseI restriction sites at the 5′ and 3′ end, respectively, was generated. The construct AAVS1-Puro-CAG-fl-STOP-fl-Cas9 was linearized with MluI (NEB, R3198S) and FseI (NEB, R0588S) to remove the Cas9 coding sequence, which was then replaced with the fragment encoding ZEB2-GFP-Flag-WPRE-poly(A) by ligation with T4 ligase. The *ZEB2* ORF was verified after every cloning step by sequencing using primers ZEB2_Seq_1-9. For simplicity, this overexpressing construct is referred to as ZEB2-GFP throughout the text. For CRISPR-Cas9 knockout of *ZEB2* in Human ESCs guide RNAs were designed using the online tool from the lab of Feng Zhang and the sequences are listed in Table S1 as ZEB2*_*sgRNA_1 & _2. Cloning of the guides was performed as outlined by Ran et al. (2013); briefly, the sense and antisense strand oligos for ZEB2_sgRNA_1 & 2 were annealed and phosphorylated, and the duplexes were cloned into pSpCas9n (BB) (PX460) and pSpCas9(BB)-2A-GFP (PX458) (a gift from Dr Fang Zhang, Addgene plasmid #48873 and #48138, respectively) by BbsI (NEB,R3539S) digestion and ligation with T4 ligase. Colonies were sequence validated using the U6_F primer.

#### Transgenic cell lines

For establishment of the H9 *ZEB2*^*+/−*^ line, plasmids pSpCas9n(BB)-*ZEB2*-Guide-A &-B (0.5 μg of each plasmid) were electroporated into 1x10^6^ H9 cells using the Human Stem Cell Nucleofector Kit 1 (Lonza, VPH-5012). Following electroporation, cells were grown in one well of a 24-well plate, reduced to a single-cell suspension and seeded into a 96-well plate at a density ranging between 1000 – 20 c/w in mTesR™1 supplemented with 1 nM ROCK inhibitor (BD Biosciences, 562822). Alternatively, cells were seeded at a density of 0.5 c/w in StemFlex supplemented with RevitaCell supplement (Thermo Fisher, A2644501). Once the cells reached ∼80% confluence the 96-well plate was split to two replica plates, one used for screening by ddPCR and the other used for further expansion. Mutant screening relied on a droplet digital PCR (ddPCR) drop-off assay (Findlay et al., 2016). Screening was performed as described in the *‘*Digital droplet PCR (ddPCR)’ section of the methods. The mutant colonies were characterized by sequencing following cloning of the edit-sites into the pJET1.2 vector (using primers ZEB2_Cas9screening_F & _R), PCR followed by DNA-PAGE (using primers ZEB2_DNAPAGE_ F & _R), karyotype analyses (outsourced to Cell Guidance Systems) and RT-PCR of pluripotency markers (details in the ‘PCR analysis’ section of the methods).

For establishment of the H9 doxycycline-inducible ZEB2 line (shortened to Hum^*iZEB2*^) and the *ZEB2*^*+/−*^*; iZEB2* rescue line, plasmids AAVS1-Neo-TRE-CMV-Cre-rtTA (2 μg), AAVS1-Puro-CAG-fl-STOP-fl-ZEB2-GFP-Flag-WPRE-poly(A) (2 μg) and PX458-AAVS1 (3 μg) were electroporated into 1.3x10^6^ H9 cells or H9 *ZEB2*^*+/−*^ cells using the Human Stem Cell Nucleofector Kit 1 (Lonza, VPH-5012). Following electroporation the cells were seeded across 3-wells of a 6 well plate coated with Matrigel in StemFlex supplemented with RevitaCell supplement (Thermo Fisher, A2644501). After 3 days from electroporation the cells were selected with StemFlex supplemented with G418 (25 μg/ml) and Puromycin (0.5 μg/ml). After approximately two weeks in selection medium, single colonies were picked and screened using three primers pair combinations; AAVS1_F & _R (AMP_1 in Figure S6), AAVS1_F & Puro_R (AMP_2 in Figure S6), AAVS1_F & Neo_R (AMP_3 in Figure S6), listed in Table S1. Individual colonies of the Hum^*iZEB2*^ were tested for *ZEB2-GFP* transgene induction by western blot and immunofluorescence following treatment with StemFlex supplemented with 1.7 μg/ml Doxycyline for 6 days.

#### Cerebral organoid generation

Cerebral organoids with telencephalic identity from human and ape cells (all female) were generated using a previously described modified protocol (Lancaster et al., 2017) to generate EBs with increased surface area:volume either through the use of fibrous microscaffolds or fewer cell numbers to generate smaller EBs. For the smaller EB approach, Aggrewell 800 plates (StemCell Technologies, 34815) containing 300 microwells, each 800 μm in size, per well were used to reproducibly generate organoids in multiples of 300. Following previously described directions for EB generation in Aggrewell plates (StemCell Technologies Document #DX21397), 6 × 10^5^ cells in 1.5 mL media were plated per well, i.e., 2,000 cells per EB. STEMdiff Cerebral Organoid Kit (StemCell Technologies, 08570) was used for organoid culture, with the addition of 1:1000 Fungizone and 1:100 Penicillin-streptomycin to avoid contamination risk associated with repeated plate usage. The timing of the protocol (StemCell Technologies Document #DX21849) was followed, with the exception of chimpanzee EBs being moved to neural induction media 2 days earlier than described. Organoids were fed every 2 days during the Aggrewell period prior to Matrigel embedding, using an electronic pipette set to the slowest speed in order to avoid flushing EBs out of microwells. Matrigel embedding was based on a previously described method (Qian et al., 2018) with minor modifications. Briefly, organoids were removed from their well, resuspended in 300 μL Expansion media and split into 3 tubes where 150 μL Matrigel was added resulting in a 3:2 Matrigel dilution. The contents of each tube were then spread into a well of an ultra-low attachment 24-well plate (Corning, 7524). After a 30-minute incubation at 37°C, 1 mL expansion media was added per well. The fibrous microscaffold approach and in-house media was used to generate *ZEB2*^*+/−*^, *ZEB2*^*+/−*^*; iZEB2*, and control. All comparisons between samples and treatments were performed on organoids generated using identical protocols.

To achieve sparse labeling of neural progenitor cells, 5 μL CytoTune emGFP Sendai fluorescence reporter (Thermo Fisher, A16519) was added to Aggrewells when the organoids were switched to neural induction medium. For ZEB2 overexpression, organoids generated from the H9 doxycycline-inducible ZEB2 (Hum^*iZEB2*^) cell line were treated with 1.7 μg/ml doxycycline (Merck, PHR1145) during the first 5 days of the protocol. For ZEB2 rescue, *ZEB2*^*+/−*^ *;iZEB2* organoids were treated with 1.7 μg/ml doxycycline from day 2 until day 7. For the pharmacological rescue of *ZEB2*^*+/−*^ organoids, starting from day 2, after embedding, *ZEB2*^*+/−*^ and WT control organoids were treated with 0.5 μg/ml Noggin (R&D, 6057-NG) and 10 μM SB-431542 (R&D, 1614) for 10 days and samples were collected for biochemical and histological analysis on day 12. For treatment with LPA, 3 μM LPA (Tocris, 325465-93-8) was added to the media of aggrewell organoids 1 day after Matrigel-embedding (3 days post neural induction, using the STEMdiff Cerebral Organoid Kit). Media was changed 2x daily, as previous studies have reported that LPA activity in culture medium decreases after 10 hours (Medelnik et al., 2018), and organoids were fixed for analysis after 48 hours of treatment (5 days post neural induction). For treatment with BMP4, 500ng/ml BMP4 (R&D, 314-BP) was added to the media of aggrewell organoids 1 day post neural induction and media was changed daily until organoids were fixed for analysis on day 5 post neural induction.

#### Histological and immunohistochemical analysis

Organoids were fixed in 4% PFA either overnight at 4°C or at room temperature for 20-60 min, and washed in PBS (3x10 min). Samples for cryostat processing were incubated overnight in 30% sucrose in 0.2 M PB (21.8 g/l Na_2_HPO_4_, 6.4 g/l NaH_2_PO4 in dH_2_O), embedded in gelatin (7.5% gelatin, 10% sucrose in 0.2 M PB), plunge frozen in 2-methylbutane (Sigma-Aldrich, M32631) at ∼-40°C for 1-2 minutes and cryosectioned at a thickness of 20 μm. Both sectioned and whole mount samples were stained as previously described (Lancaster et al., 2013). For histological analysis, sections were stained for hematoxylin and eosin (Carl Roth, 9194.1) followed by dehydration in ethanol and xylene and mounting in permount mounting media.

#### Imaging and image analysis

Confocal images of fixed cryosectioned samples were obtained with a Zeiss LSM 780 or 880 confocal microscope. Images of fixed whole mount samples were obtained with a Zeiss LSM 780 confocal microscope or a Nikon CSU-W1 spinning disk microscope. Whole mount organoids were imaged at 8 μm intervals on Zeiss LSM 780 while the speed of the Nikon CSU-W1 system allowed for higher resolution imaging at 0.5 μm intervals. To calculate the apical luminal surface area of organoids, folders containing Zeiss LSM 780 Z stacks of ZO1 stained organoids as an image stack were carried over to MATLAB (Mathworks). The largest lumen per organoids was segmented using a tailor-made code that delineates the perimeter of the lumen in each Z stack by detecting fluorescent intensity and then combines the delineated perimeters into a 3D structure from which the surface area is calculated. This code is publicly available (https://github.com/esriis/organoid-lumen-segmentation). The apical surface area of individual cells was measured from cryosections of organoids where cells were selected based on SOX2+ staining and sectioning angle being at the apical surface of progenitors. ZO1 stain was used as a marker of apical cell perimeters and Fiji (Schindelin et al., 2012) was used to manually delineate perimeters and perform apical surface area measurements. Because of differences in tissue shrinkage during PFA fixation and handling, comparisons were performed with controls fixed and stained at the same time and treated identically. TBR2 positive cells (Figure S6T) were counted using a FIJI plug-in generated by Johannes Schindelin. For quantification of TBR2+ cell numbers per unit area in ZEB2-rescue and day 55 organoids, a custom Fiji macro was used to count the number of TBR2+ cells, this was then divided by the corresponding area. This macro is publicly available (https://github.com/StefanoLG92/FIJI-macros). Distance of nuclei as a percentage of VZ length was performed in Fiji on cryosections of organoids stained with DAPI and ZO1, where ZO1 staining indicated the sectioning angle was perpendicular to the apical surface of neuroepithelial tissue. The distance from the ZO1+ apical surface to the center of nuclei was measured and reported as a percentage of the total apico-basal thickness of the neuroepithelial tissue. Total thickness was measured from ZO1+ apical surface to the basal surface as determined by background low-level fluorescence from the tissue (average of 3 total thickness measurements). To segment and quantify apical processes of individual neural progenitor cells, the surfaces option in Imaris (Oxford Instruments, v9.2) was used to manually segment virally-labeled GFP+ cells that were clearly separated from neighboring labeled cells using images of whole mount organoids obtained on a Nikon CSU-W1 spinning disk microscope. To capture differences in apical process thickness, apical process volumes were derived by cutting segmented cells apical to the cell body (‘cut surface’ in Imaris) and obtaining the volume of the isolated apical processes in Imaris. Because volume can be influenced by topology (i.e., curvature, length), in ways that do not necessarily reflect thickness (diameter), we normalized volume measurements to surface area to account for these topological effects.

For quantification of organoid size and neuroepithelial bud perimeter, brightfield images of live organoids were taken on an EVOS (Thermo Fisher, AMF4300). Organoid area was measured by manually delineating the perimeter of individual organoids in Fiji and measuring the area within these structures. Neuroepithelial bud perimeters were obtained by manually delineating the visible basal surface of neuroepithelial buds projecting from the main organoid mass and measuring their lengths.

#### Immunoblotting

Cell and organoid samples were washed twice in ice-cold PBS, pelleted by centrifugation (500 g, 3 min) and lysed with modified-RIPA (mRIPA: 1% Triton-X, 0.1% SDS, 150 mM NaCl, 50 mM Tris pH 7.4, 2 mM EDTA, 12 mM sodium deoxycholate) supplemented immediately prior to lysis with protease (Thermo Fisher, 78430) and phosphatase (Sigma-Aldrich, 4906845001) inhibitors. The protein concentration of the samples was measured using the Quick Start Bradford Dye Reagent (Bio-Rad, 5000205). Between 3-20 μg of total protein per sample were resolved by SDS-PAGE (4%–20% gels) and transferred to Amersham Hybond P 0.45 PVDF blotting membranes (GE Healthcare, 10600023). Membranes were blocked overnight at 4°C in 5% milk or 5% BSA in PBST. Specific blocking conditions were optimized for each antibody during the initial validation stages. Primary antibodies were incubated overnight at 4°C. HRP-linked goat anti-rabbit (Dako, P0448, 1:3000) and rabbit anti-mouse (Dako. P0161, 1:3000) secondary antibodies were incubated for ∼1 hr at room temperature. The blots were developed using ECL Prime enhanced chemoluminescent detection reagent (GE Healthcare, RPN2232) or alternatively SuperSignal West Femto chemoluminescent substrate (Thermo Fisher, 34094) and X-ray films (Photon Imaging Systems Ltd, FM024) and developer or a Gel Doc XR^+^ system.

#### Antibodies

Primary antibodies used for protein detection, with their corresponding dilutions for immunofluorescence (IF), western blotting (WB) and WB blocking conditions were as follows: mouse anti-β-actin (Abcam, 8226, WB 1:2000 in BSA), mouse anti-ZEB2 (Origene, TA802113, IF 1:150, WB 1:2000 in milk), sheep anti-TBR2 (R&D Systems, AF6166, IF 1:200), mouse anti-CDH2 (BD Biosciences, 610920, IF 1:500, WB 1:1000 in milk), mouse anti-CDH1 (BD Biosciences, 610181, IF 1:500, 1:1000 in milk), rabbit anti-OCLN (Abcam, ab31721, IF 1:200, WB 1:1000 in milk), rabbit anti-EMX1 (ATLAS Antibodies, HPA006421, IF 1:100), rabbit anti-EMX1 (Origene, TA325087, WB 1:1000 in BSA), rabbit anti-BLBP (Abcam, ab32423, IF 1:200), rabbit anti-GLAST (Abcam, ab416, IF 1:200), goat anti-DCX (N-19) (Santa Cruz, sc-8067, IF 1:300), rat anti-CTIP2 (Abcam, ab18465, IF 1:200), mouse anti-HuC/D (Life Technologies, A21271, IF 1:200), mouse anti-ZO1 (BD Biosciences, 610966, IF 1:300), chicken anti-GFP (Thermo Fisher, A10262, IF 1:500), rabbit anti-GFP (Abcam, ab290, WB 1:1000 in milk), rabbit anti-EpCAM (Abcam, ab71916, IF 1:300, WB 1:1000 in milk), mouse anti-Vimentin (V9) (Santa Cruz, sc-6260, IF 1:200, WB 1:1000 in BSA) rabbit anti-PAX6 (Abcam, ab195045, IF 1:200), rabbit anti-SOX2 (Abcam, ab97959, IF 1:200), rabbit anti-SHROOM3 (ATLAS Antibodies, HPA047784, IF 1:200, WB 1:1000), mouse anti-GAPDH (Abcam, ab8245, WB 1:2000 in milk), mouse anti-TUJ1 (Biolegend, 801213, IF 1:500). Alexa Fluor 594 Phalloidin (Thermo Fisher, A12380, 1:400) was used to detect F-actin. Alexafluor 405, 488, 568 and 647 secondary antibodies (Thermo Fisher) were used for detection of primary antibodies in IF.

#### PCR analysis

For applications aimed at amplicon size comparison GoTaq Green Master Mix (Promega, 9PIM712) was used according to the manufacturer’s guidelines. For molecular cloning or any other application requiring high sequence fidelity Q5 High Fidelity 2X Master Mix (NEB, M0492S) was used. PCR analysis of pluripotency markers was done using the Human Pluripotent Stem Cell Assessment Primer Pair Panel (R&D Systems, SC012) and amplification was done using GoTaq Green. Novex TBE 10% gels (Thermo Fisher, EC6275BOX) were used for DNA-PAGE analysis of *ZEB2*^+/−^ mutant gDNA. The primers used were ZEB2_DNAPAGE_F & R (Table S1), GoTaq green was used for amplification and 1X SYBR Gold Nuclei Acid Stain (Thermo Scientific, S11494) was used for detection. Samples were prepared as outlined in the PAGE gels technical sheet.

#### Droplet digital PCR (ddPCR)

In order to detect CRISPR mutants and perform enrichment by sib-selection a TaqMan-based ddPCR drop-off assay was designed (Findlay et al., 2016). An amplicon of 198 bp overlapping the edited genomic region (GRCh38/hg38 chr2:144,517,275-144,517,351) was produced using primers ZEB2_Cas9screening_F & R. The assay was designed so as to have a 5′-HEX-labeled 3′-BHQ1 probe binding to a nick site (i.e., ZEB2_drop-off_probe) and a 5′-FAM-labeled 3′-BHQ1 probe binding to both edited and WT amplicons (i.e., ZEB2_reference_probe). The specific sequence of primers and probes used in the assay are listed in Table S1. The assay was performed using the ddPCR Supermix for Probes (Bio-Rad, 1863024) as described in the product’s technical bulletin. Briefly, reaction mixes (20 μl/reaction) were prepared as follows: 100 nM primers, 200 nM probes, 10 U MseI (NEB, R0525S), 1x ddPCR supermix for probes and 50-300 ng of genomic DNA (gDNA). The reactions were loaded into DG8 cartridges (Bio-Rad, 1864008) with droplet generation oil for probes (Bio-Rad, 1863005), the cartridge was then fitted with the DG8 gaskets (Bio-Rad, 1863009) and run on the QX200 droplet generator (Bio-Rad, 10031907). The droplet-oil emulsions were transferred to a ddPCR-compatible 96-well plate that was sealed with the PX1 PCR Plate Sealer (Bio-Rad) and the PCR reaction was run on a C1000 touch thermal cycler following the PCR protocol detailed in the technical bulletin. After thermal cycling, data were acquired on the QX200 Droplet Reader (Bio-Rad) using the QuantaSoft Software (Bio-Rad). Negative and positive control samples for the assay were gDNA extracts from HEK293 cells and HEK293 cells transfected with plasmids pSpCas9(BB)-2A-GFP-*ZEB2-*guide-A & -B, respectively.

#### RNA-seq sample preparation and sequencing

Organoids generated from 3 replicate batches of H9 and G1 cell lines were collected at 7 time points for RNA-seq analysis: 0, 2, 3, 5, 10, 15 and 25 days post neural induction. ∼300 organoids per replicate were collected at the time points ranging between day 0 and 5, ∼150 organoids at day 10, ∼100 organoids at day 15, and ∼50 organoids at day 20. Organoids were collected in Eppendorf tubes, washed with PBS, flash-frozen in liquid nitrogen and stored at −80°C.

RNA from all 42 samples was isolated in parallel with the Direct-zol-96 RNA kit (Zymo Research, R2055), following the product’s manual. cDNA libraries were generated according to NEBNext Ultra II Directional RNA Library Prep Kit for Illumina (NEB E7760). cDNA was amplified for 12 cycles and barcoded with NEBNext Multiplex Oligos for Illumina (NEB E7600). Libraries were tested for quality using the 2100 Bioanalyzer system (Agilent) and concentrations were measured using Qubit dsDNA HS Assay Kit (Thermo Fisher, Q32851) on Qubit 3 Fluorometer (Thermo Fisher). Samples were pooled together to 15 nM concentration and sequenced with single-end 50 base mode on 3 lanes of an Illumina HiSeq 4000 instrument.

#### RNA-seq data analysis

RNA-seq reads were processed using the PRAGUI pipeline (https://github.com/lmb-seq/PRAGUI). Reads were trimmed using Trim Galore! version 0.6.3_dev (Krueger, 2012) (https://github.com/FelixKrueger/TrimGalore) with Cutadapt version 2.4 (Martin, 2011). Default parameters were used. Read quality was analyzed by FASTQC version v0.11.5 (Andrews, 2010). Human and gorilla reads were aligned to GRCh38 and Kamilah_GGO_v0 reference genomes respectively using HISAT2 v2.0.0-beta (Kim et al., 2015). GTF gene annotation files for both of the reference genomes were downloaded and filtered for a list of 16,763 genes that are annotated with identical gene names in both species. Reads were assigned to genes in the custom GTF files and counted using HTSeq version 0.11.2 (Anders et al., 2015). Read counts were then normalized to transcripts per million (TPM) within each sample and log2 transformed (log2(TPM+1)). Unbiased clustering of the samples with principal component analysis was performed using the 3000 most variable genes. Log2 transformed TPM values were converted to z-scores, (x_i_ – μ)/σ, where x_i_ is the TPM at a given time point, μ is the mean TPM of the gene across all time points and σ is the standard deviation of the TPM across all time points. Pearson correlation was performed on z-scaled mean log2 transformed TPM and on all replicates z-scaled. Hierarchical clustering was done on z-scaled means of the 3000 most variable genes of mean log2 transformed TPMs. GO term enrichment analysis was performed using the online g:Profiler software (Reimand et al., 2007) (https://biit.cs.ut.ee/gprofiler/gost).

#### Differential temporal expression analysis

RNA-seq analysis was performed using R v3.5.3 (R Core Team, 2018). To assess temporal changes in gene expression between day 3, 5 and 10, the list of 16,763 genes was first filtered based on the following criteria: 1) genes were expressed at > 10 TPM in at least one time point in all replicates of both species, 2) genes displayed a fold-change of > 1.5 TPM between any two time points in at least one species. This resulted in a list of 3,526 genes. Log2 normalized TPMs were then z-scaled across the three time points. Next, the gene list was further filtered in order to remove genes with variable expression patterns across replicates by removing genes with a squared difference > 6 in either species. The squared difference value was obtained by calculating the squared difference of z-scores per time point between all three replicates and then taking the sum of this number for all time points. This list of 2,905 genes was used for time course sequencing data analysis using the TCseq package (Wu and Gu, 2020) available online (https://rdrr.io/bioc/TCseq/f/inst/doc/TCseq.pdf). Replicates were kept separate for the analysis, meaning that the input was 17,430 unique patterns of gene expression representing 2,905 genes in 2 species and 3 replicates per species. The TCSeq analysis was run using the *timeclust* function with the settings *algo = ‘cm’, k = 10*, resulting in the unsupervised soft clustering of gene expression patterns into 10 clusters with similar z-scaled temporal patterns. Replicates of 563 genes were found to be in 3 different clusters in at least one species and were removed from downstream analysis resulting in 2,342 genes (Data S1). 22.5% of genes, 527 genes, were found to never be in the same cluster between species showing a robustly different expression pattern (Data S1). Genes were assigned to the cluster where 2 or all of the replicates were found per species. 59% of genes were assigned to different clusters between species. GO term enrichment analysis was performed on the genes present in each of the 10 clusters generated by TCseq per species, resulting in a total of 159 GO:BP terms found in both species, of which 85 were found to be moving between species (Data S1). GO term analysis on the 527 genes moving robustly between species revealed 67 were linked to enriched cell morphogenesis-related terms (“cell morphogenesis,” “cell part morphogenesis,” “cellular component morphogenesis”). These genes were intersected with a list of 1,639 confirmed transcription factors (Lambert et al., 2018), revealing 8 transcription factors related to cell morphogenesis (Figure 5F).

#### Live imaging and cell cycle analysis

For live imaging, a SiMView light sheet microscope was primarily used in addition to an Andor Revolution spinning disk microscope. Whole organoids were imaged on a SiMView light-sheet microscope with environmental controls as described in McDole et al. (2018). Briefly, organoids were embedded in Matrigel inside of a glass capillary or placed on a cylindrical PEEK platform and either extruded into or placed inside a Teflon FEP tube (Zeus, Inc.) filled with expansion media. This was then placed inside the imaging chamber, which was kept at 37°C and 5% CO2 in atmosphere using an Okolab Bold line environmental system. Culture media was exchanged for fresh media every 24 hours. Image stacks were acquired using a 488 nm laser every 5 minutes with 2.031 μm z-spacing using two Nikon 16x 0.8 NA water-dipping objectives for detection and two Special Optics 54-12.5-31 @ 450-1000nm 6.45x 0.2NA illumination objectives. Images were collected by two Hamamatsu Orca Fusion cameras. Acquired images were then processed according to previously described methods (Amat et al., 2015; McDole et al., 2018). For analysis of cell cycle duration, individual cells were tracked manually using the MaMuT plugin in Fiji (Wolff et al., 2018) and the cell-cycle calculated as the time period between two divisions. For imaging on the Andor Revolution spinning disk microscope, organoids were placed on an imaging dish (2BScientific, 6160-30) and were held in place by a drop of Matrigel that was allowed to polymerize at 37°C before addition of expansion media. Single cells were selected as the focal point and a images were acquired at 4% laser power every 20 minutes and stacked at 2 μm intervals in an incubation chamber set to 37°C and 5% CO_2_ between day 3 and 5 post neural induction.

#### Growth curve modeling

Mathematical modeling of progenitor growth used a standard growth curve equation:

$$p_{t}=p_{t-1}2^{f}$$

where $p_{t}$ is the number of progenitors at time t in days, and f is the number of divisions in 1 day. This equation was applied to calculate the number of progenitors in human and gorilla using arbitrary time length and starting cell number. There were certain assumptions governing the shape of the curves, but these assumptions were applied equally to both the human and gorilla. These assumptions were: 1. Divisions were symmetric expansive for the first half of the time period, with f being higher early and lower at later time points. 2. During the second half of the time period, divisions were increasingly asymmetric neurogenic, where proportions of symmetric to asymmetric were taken from *in vivo* measurements (Noctor et al., 2008).

Because of the second assumption, during the second half of the time period, the growth curve was modified as follows:

$$p_{t}=\left(p_{t-1}r_{a}\right)+\left(p_{t-1}r_{s}2^{f}\right)$$

where $r_{a}$ is the ratio of divisions that are asymmetric at time t and $r_{s}$ is the ratio of divisions that are symmetric at time t.

Number of neurons was calculated from the progenitor number at each time point according to the following equation:

$$n_{t}=n_{t-1}+\left(p_{t-1}r_{a}\right)$$

where $n_{t}$ is the number of neurons at time t in days, and $p_{t-1}$ is the number of progenitors at t−1 as calculated above.

The only difference between human and gorilla models was an increased f in human for a period of 5 days during the symmetric expansive phase, which was the length of time in which human and gorilla exhibited differences in their cell morphology (day 3 to day 8, Figure 3B). During this time period, $f_{human}$was equal to 1.274, calculated from the measured average cell cycle length of 18.831 hours, while $f_{gorilla}$was equal to 1.086, calculated from the measured average cell cycle length of 22.099.

Overall, the model revealed an increase in human of 1.9 fold in both $p_{t}$ and $n_{t}$, which was consistent and independent of the arbitrary values otherwise used to generate the growth curves.

### Quantification and statistical analysis

All biological experiments were performed with multiple independent biological samples as detailed in the figure legends. Numbers of samples are detailed in figure legends for each quantification performed, including numbers of cells, organoids, and batches for each cell line. Statistical test details can be found in the figure legends. For comparisons across multiple samples, multiple comparisons corrections were applied as detailed in figure legends. Statistics were performed and graphs were generated in Prism v8.4.2 (Graphpad).

## Supplemental information

Table S1. Table of oligonucleotides, related to STAR Methods Sequence and application of oligonucleotide primers.

Data S1. Data related to interspecies comparisons from RNA-seq data analysis and GO term enrichment analysis, related to Figures 4 and 5
