# Supplementary material for: An early cell shape transition drives evolutionary expansion of the human forebrain
Source: Cell. 2021 Apr 15;184(8):2084–2102.e19. doi: 10.1016/j.cell.2021.02.050 (PMC8054913; doi:10.1016/j.cell.2021.02.050)
Supplement: Table S1. Table of oligonucleotides, related to STAR Methods — Sequence and application of oligonucleotide primers. [file mmc1.pdf]

Cell, Volume *184*

Supplemental information

An early cell shape transition drives evolutionary expansion of the human forebrain

Silvia Benito-Kwiecinski, Stefano L. Giandomenico, Magdalena Sutcliffe, Erlend S. Riis, Paula Freire-Pritchett, Iva Kelava, Stephanie Wunderlich, Ulrich Martin, Gregory A. Wray, Kate McDole, and Madeline A. Lancaster

Supplemental Table 1. Table of Oligonucleotides, Related to STAR Methods

Name	Sequence (5'-3')	Application
AAVS1_CAG_fl_STOP_fl_F	ACGCGTAGTCGGTACCTTACTAGGGACAGGATTGGTGACAG	Amplification of plasmid backbone for construction of AAVS1-Puro-CAG-fl-STOP-fl-Cas9
AAVS1_CAG_fl_STOP_fl_R	CTCGACCCATAGAGCCCAC	Amplification of plasmid backbone for construction of AAVS1-Puro-CAG-fl-STOP-fl-Cas9
Cas9_β_globin_pA_F	ATAACGCGTATGGACTATAAGGACCACGACGG	Amplification of Cas9-b-globin-poly(A) and addition of resriction sites
Cas9_β_globin_pA_R	ATAGGTACCATTTGCCCTCCCATATGTCCTTC	Amplification of Cas9-b-globin-poly(A) and addition of resriction sites
TRE_F	(P)-CTAGTAAAGCTTAGTACTGTCGAGTTTAC	Amplification of tight TRE promoter
TRE_R	(P)-TTAGTCGACACAGGCGATCTGACGGTTC	Amplification of tight TRE promoter
CMV_bGH_F	(P)-GCTACAACAAGGCAAGGCTTGACC	Amplification of CMV-2TO-MCS-bGHpoly(A)
CMV_bGH_R	(P)-TTCTTTCCGCCTCAGAAGCCATAG	Amplification of CMV-2TO-MCS-bGHpoly(A)
Cre_SalI_F	TTGTCGACGCAACGTGCTGGTTATTGTGCTG	Amplification of Cre
Cre_KpnI_R	ATGGTACCTCATCCATCGCCATCTTCCAGCAG	Amplification of Cre
AAVS1_F	CTGCCGTCTCTCTCCTGAGT	Genotyping of the *AAVS1* locus
AAVS1_R	CTGGCTCCATCGTAAGCAAACC	Genotyping of the *AAVS1* locus
Puro_R	AGAGTTCTTGCAGCTCGGTGAC	Genotyping of the edited AAVS1 locus
Neo_R	AGCAGCCGATTGTCTGTTGTG	Genotyping of the edited AAVS1 locus
AAVS1_sgRNA	GGGGCCACTAGGGACAGGA	sgRNA used to target the AAVS1 locus
ZEB2_IndOE_F	AACGTGCTGGTTATTGTGCTGTCTCATCATTTTGGCAAAGATGAAGCAGCCGATCATGGCGGA	Amplification and tagging of the *ZEB2* ORF
ZEB2_IndOE_R	CACCGCCACCGGATCCACCGCCACCGGATCCACCGCCACCCATGCCATCTTCCATATTGTCTTCCT	Amplification and tagging of the *ZEB2* ORF
GFP_IndOE_F	GGTGGCGGTGGATCCGGTGGCGGTGGATCCGGTGGCGGTGGATCCATGGTGAGCAAGGGCGAGGA	Amplification and tagging of the *ZEB2* ORF
GFP_IndOE_ R	CTCTCCGCTGCCAGAACTCTCGAGGCCCTGTGGACGCGTACCGGTCTTATCGTCGTCATCCTTGTAATCGGATCCACCGCCACCCTTGTACAGCTCGTCCATGCCG	Amplification and tagging of the *ZEB2* ORF
ZEB2_GFP_Flag_ IndOE_F	ATACGCACCGGTATGAAGCAGCCGATCATGGCG	Amplification of ZEB2-GFP-Flag for subcloning
ZEB2_GFP_Flag_ IndOE_R	ATACGCGGTACCTTACTTATCGTCGTCATCCTTGTAATCGGATCC	Amplification of ZEB2-GFP-Flag for subcloning
ZEB2_AAVS1 IndOE_F	ACATGCACGCGTATGAAGCAGCCGATCATGGCG	Amplification of ZEB2-GFP-Flag-WPRE-poly(A) for construction of the AAVS1-Puro-CAG-fl-STOP-fl-ZEB2-GFP-Flag-WPRE-poly(A) construct
ZEB2_AAVS1 IndOE_R	ACATGCGGCCGGCCCCGAGTGAGAGACACAAAAAATTCCAACA	Amplification of ZEB2-GFP-Flag-WPRE-poly(A) for construction of the AAVS1-Puro-CAG-fl-STOP-fl-ZEB2-GFP-Flag-WPRE-poly(A) construct
ZEB2_Seq_1	GCATGACCATCGCGTTCCTC	Sequence validation of the *ZEB2* ORF
ZEB2_Seq_2	CACCCCTGGCACAACAAC	Sequence validation of the *ZEB2* ORF
ZEB2_Seq_3	GAGGAACGCGATGGTCATGC	Sequence validation of the *ZEB2* ORF
ZEB2_Seq_4	TTTGCCTACCGCACCCAG	Sequence validation of the *ZEB2* ORF
ZEB2_Seq_5	TGCTCAGAGTCCAATGCAGCA	Sequence validation of the *ZEB2* ORF
ZEB2_Seq_6	TCAAGGCGGTCCTGCAGC	Sequence validation of the *ZEB2* ORF
ZEB2_Seq_7	AGGAGCTCCAGGCTGAGC	Sequence validation of the *ZEB2* ORF
ZEB2_Seq_8	ACAAGACTACATGTCAGGCCTAG	Sequence validation of the *ZEB2* ORF
ZEB2_Seq_9	ACGGATCCCGAAACGATACGAG	Sequence validation of the *ZEB2* ORF
ZEB2_sgRNA_1	CGGGGGCCATCCGCCATGATCGG	sgRNA used to target *ZEB2*
ZEB2_sgRNA_2	GGCGCAAACAAGCCAATCCCAGG	sgRNA used to target *ZEB2*
ZEB2_DNAPAGE_ F	GCCATCTGATCCGCTCTTAT	Amplification of a 114 bp fragment of the ZEB2 edit site for DNA-PAGE
ZEB2_DNAPAGE_ R	GGTTCGGGCTGCTTCTTAC	Amplification of a 114 bp fragment of the ZEB2 edit site for DNA-PAGE
ZEB2_Cas9screening_F	CCACATTGTCGCTGTGTTTG	Primer for TaqMan drop-off assay on *ZEB2* edits
ZEB2_Cas9screening_R	GAAAAGTTTGGTTCGGGCTG	Primer for TaqMan drop-off assay on

		ZEB2 edits
ZEB2_drop-off_probe	(HEX)-TCCTGGGATTGGCTTGTTTGCG-(BHQ1)	Probe for TaqMan drop-off assay on *ZEB2* edits
ZEB2_reference_probe	(FAM)-AGGGCGCCGAGTCCATGC-(BHQ1)	Probe for TaqMan drop-off assay on *ZEB2* edits
ZEB2_RTPCR	CGCTTGACATCACTGAAGGA	Amplification of *ZEB2* mRNA for RT-PCR analysis
ZEB2_RTPCR	GCTCCTTGGGTTAGCATTTG	Amplification of *ZEB2* mRNA for RT-PCR analysis
AAVS1_CAG_fl_STOP_fl_F	ACGCGTAGTCGGTACCTTACTAGGGACAGGATTGGTGACAG	Amplification of plasmid backbone for construction of AAVS1-Puro-CAG-fl-STOP-fl-Cas9
AAVS1_CAG_fl_STOP_fl_R	CTCGACCCATAGAGCCCAC	Amplification of plasmid backbone for construction of AAVS1-Puro-CAG-fl-STOP-fl-Cas9

Modifications:
(P) 5'-phosphorylation
(HEX) 5'-HEX fluorophore
(FAM) 5'-FAM fluorophore
(BHQ1) 3'-black hole quencher 1
